# Synthesis and crystal structure of tetra­kis­(perfluoro­benzo­nitrile)­silver(I) tetra­kis­(perfluoro-*tert*-but­oxy)aluminate(1−): a perfluorinated silver(I) nitrile cation with a perfluorinated weakly coordinating anion

**DOI:** 10.1107/S205698902600976X

**Published:** 2026-09-30

**Authors:** Eric Wu, James D. Watson, Ruoming Tian, Mohan Bhadbhade, Graham E. Ball

**Affiliations:** a School of Chemistry, University of New South Wales, Sydney, NSW 2052, Australia; b Mark Wainwright Analytical Centre, University of New South Wales, Sydney, NSW 2052, Australia; University of Buenos Aires, Argentina

**Keywords:** silver complexes, nitrile ligands, perfluoro­benzo­nitrile, weakly coordinating anions, perfluorinated salts, crystal structure

## Abstract

The title compound, [Ag(C_6_F_5_CN)_4_][Al{OC(CF_3_)_3_}_4_], was obtained by reaction of Ag[Al(OC(CF_3_)_3_)_4_] with excess perfluoro­benzo­nitrile. The crystals belong to the chiral space group *P*2_1_ with *Z*′ = 2, containing two independent [Ag(C_6_F_5_CN)_4_]^+^ cations and two [Al(OC(CF_3_)_3_)_4_]^−^ anions in the asymmetric unit. Each Ag^I^ centre is four-coordinate and adopts a distorted tetra­hedral geometry. One of the nitrile ligands in both Ag^I^ centres has a noticeably longer Ag—N distance [2.383 (8) and 2.371 (9) Å] and the Ag—N—C angle [148.3 (7) and 148.8 (8)°] deviates significantly from linearity in both of these cases. The other Ag—N distances are between 2.207 (8) and 2.280 (8) Å with Ag—N—C angles between 165.4 (7) and 177.0 (7)°. N—Ag—N angles vary from 94.5 (3) to 120.3 (3)°. In the crystallographic overlay, the two Ag^I^ cations appear as approximate non-superimposable mirror images. The arrangement of the cations dominates the crystal packing with the anions occupying the spaces between the cations. There are no short Ag⋯anion contacts. The structure contains numerous short F⋯F contacts, which is unsurprising given the perfluorinated nature of the system, and there is an indication of C—F⋯π type inter­actions. ^19^F NMR spectra of the complex were consistent with the crystallographically determined structure with four perfluoro­benzo­nitrile ligands.

## Abstract 2

The synthesis and structure of a homoleptic silver(I) complex with four perfluoro­benzo­nitrile ligands bound in a distorted tetra­hedral arrangement combined with perfluoro­aluminate counter-ion are described.

## 1. Chemical context

Silver(I) nitrile complexes are well known and are often four-coordinate, with approximately tetra­hedral coordination geometries that are frequently distorted by ligand steric and packing effects. For example, the homoleptic complex [Ag(NCCH_3_)_4_]^+^ has been crystallographically characterized with a range of counter-anions, including the weakly coordinating [B(C_6_F_5_)_4_]^−^ anion (aka [BArF_20_]^−^) (CSD refcode EFAQOU; Kim *et al.*, 2017 ▸). The linear two-coordinate arrangement is also common and [Ag(NCCH_3_)_2_]^+^ has been reported with the [B{3,5-(CF_3_)_2_C_6_H_3_}_4_]^−^ anion (aka [BArF_24_]^−^) (FILTEA; Zhang *et al.*, 2005 ▸).

We were therefore inter­ested in determining whether reaction of Ag^I^ with the fluorinated aryl nitrile ligand perfluoro­benzo­nitrile (C_6_F_5_CN) would afford either a two or a four-coordinate complex. In addition to its coordination chemistry, C_6_F_5_CN offers the possibility of forming a variety of weak inter­actions between the perfluoroaryl rings in the solid state. The weakly coordinating anion [Al(OC(CF_3_)_3_)_4_]^−^ was selected to minimize cation-anion inter­actions, thereby allowing the intrinsic coordination preferences of the Ag^I^ centre and the packing behaviour of the fluorinated nitrile ligands to be examined.
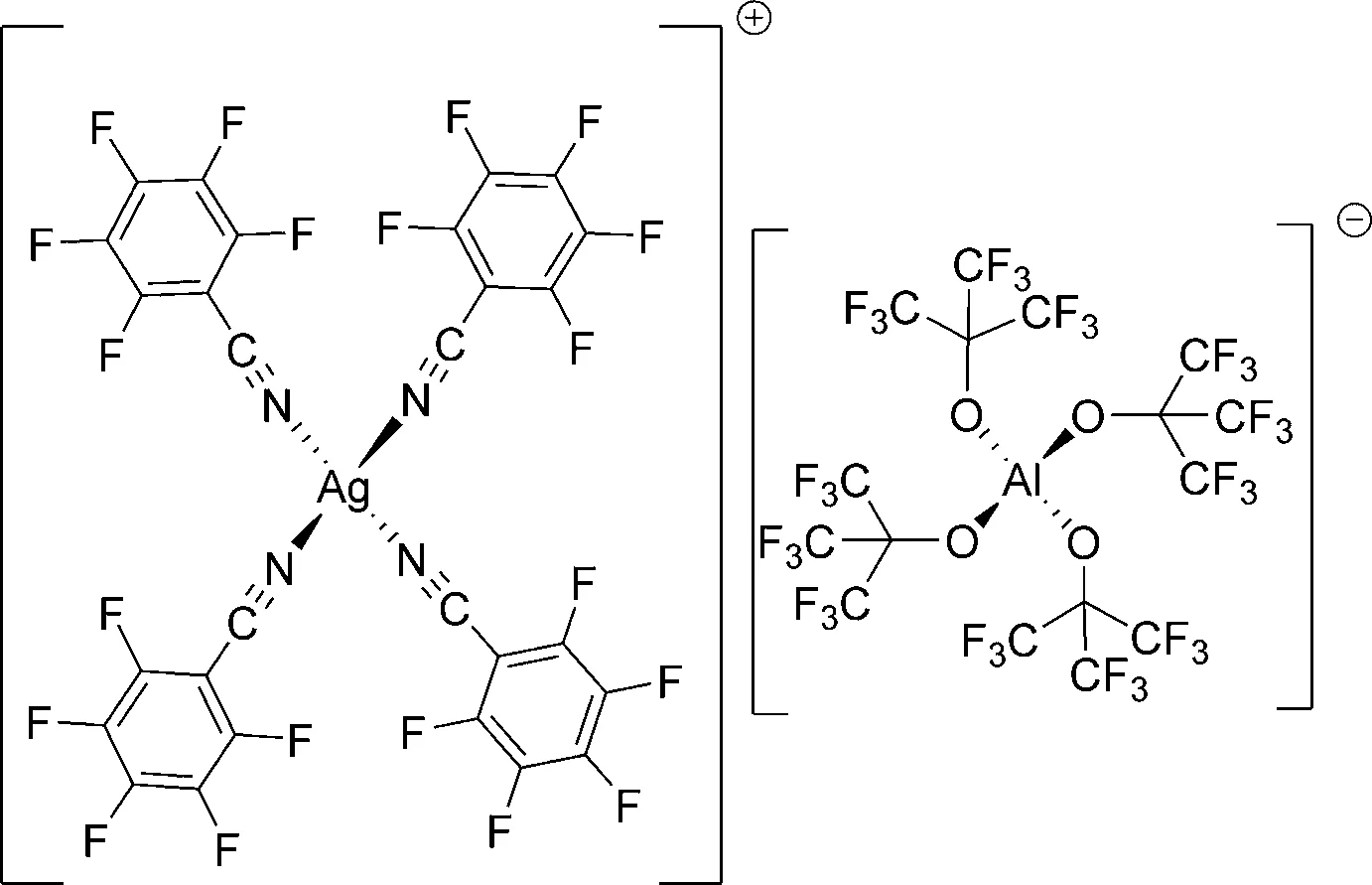


## 2. Structural commentary

The title complex crystallized in the chiral space group *P*2_1_ with *Z*′ = 2, containing two independent [Ag(C_6_F_5_CN)_4_]^+^ cations and two [Al(OC(CF_3_)_3_)_4_]^−^ anions in the asymmetric unit.

Each Ag^I^ centre coordinates four C_6_F_5_CN ligands and adopts a distorted tetra­hedral geometry. The two cations have similar coordination environments but differ slightly in conformation. In the crystallographic overlay, they appear as approximate non-superimposable mirror images. One Ag—N bond is slightly elongated, *i.e*., 2.383 (8) Å in mol­ecule *A* and 2.371 (9) Å in mol­ecule *B*, and these correspond to a more strongly bent metal-nitrile inter­action [Ag—N—C angle 148.3 (7)° in *A*, 144.8 (;8)° in *B*], whereas the remaining three Ag-N distances are shorter [2.207 (8) to 2.280 (8) Å] and have correspondingly less distorted coordination [Ag—N—C angles between 165.4 (7)deg; and 177.0 (7)°]. This raises the possibility that the nitrile ligand with the longer, more bent inter­action is less tightly bound than the other three.

Distortion from tetra­hedral coordination of the nitro­gen donors is moderate, with N—Ag—N angles varying from 94.5 (3) to 120.3 (3)° in mol­ecule *A* and 98.1 (3) and 119.7 (3)° in mol­ecule *B*. Coordination geometries of the Ag centre in mol­ecule *A* and mol­ecule *B* are given in Fig. 1 ▸.

There are no observable short contacts between either Ag^I^ centre and atoms of the anions.

## 3. Supra­molecular features

The overlay of complex cations *A* and *B* highlights their conformational relationship (Fig. 2 ▸*a*). The packing of mol­ecules (Fig. 2 ▸*b* and Fig. 3 ▸) in the crystal is dominated by the arrangement of the complex cations, with the anions occupying the voids between them. The structure also exhibits numerous short F⋯F contacts, which are common in perfluorinated systems and contribute to the packing arrangement. Fig. 2 ▸*b* highlights apparent C—F⋯π inter­actions [C3*H*—*Cg*1: C⋯*Cg* = 3.548 (7) Å, F⋯*Cg* = 4.809 (10) Å, C—F⋯*Cg* = 155.6 (5)° and C5*D*—*Cg*2: C⋯*Cg* = 3.527 (7) Å, C—F⋯*Cg* = 4.771 (10) Å, C—F⋯*Cg* = 156.3 (5)° where *Cg*1 and *Cg*2 are the centroids of the C1*C*–C6*C* and C1*G*–C6*G* rings, reespectively] that are involved in creating a pseudo-centre of inversion between the two cations.

## 4. Database survey

A search of the CSD (version 6.01, May 2026 update; Groom *et al.*, 2016 ▸) for homoleptic nitrile complexes of silver(I) paired with the [Al(OC{CF_3_)_3_}_4_]^−^ anion indicates that with the tri­chloro­aceto­nitrile ligand, CCl_3_CN, both four coordinate [Ag(NCCCl_3_)_4_]^+^ (UDOYUK; Engesser *et al.*, 2018 ▸) and two coordinate [Ag(NCCCl_3_)_2_]^+^ (EKUPEI; Malinowski *et al.*, 2021 ▸) complexes are known, the latter being formed presumably by removal of CCl_3_CN ligands under vacuum (Malinowski *et al.*, 2021 ▸). Crystallographically characterized complexes of the fluorinated aceto­nitrile ligands CH_2_FCN and CHF_2_CN are also two-coordinate, [Ag(NCCH_2_F)_2_]^+^ (LIGLEW) and [Ag(NCCHF_2_)_2_]^+^ (LIGLAS; Jadwiszczak & Malinowski, 2023 ▸). Again, both two-coordinate compounds with fluorinated aceto­nitrile ligands were exposed to high vacuum prior to their crystallization, which may encourage lower coordination numbers. Only one homoleptic perfluoro­benzo­nitrile complex cation was found, the octa­hedral zinc complex [Zn(NCC_6_F_5_)_6_][AsF_6_] (JEPKUL; Akkuş *et al.*, 2006 ▸).

## 5. Synthesis and crystallization

Perfluoro­benzo­nitrile (C_6_F_5_CN) and penta­fluoro­benzene were dried over 3 Å mol­ecular sieves and degassed using three freeze-pump-thaw cycles on a Schlenk line prior to use. Ag[Al{OC(CF_3_)_3_}_4_] was prepared according to literature methods (Krossing, 2001 ▸).

Ag[Al{OC(CF_3_)_3_}_4_] (0.200 g, 0.186 mmol) was dissolved in penta­fluoro­benzene (3 ml) under argon at room temperature. C_6_F_5_CN (1.11 mmol, 0.14 ml, 5.9 equivalents) was then added slowly to the solution and stirred for approximately 10 minutes. The complex was crystallized by vapour diffusion of pentane into the penta­fluoro­benzene solution over the course of 3 days. The reaction vessel was covered with aluminium foil throughout this experiment to reduce light exposure.

After three days, small, colourless, clear crystalline plates had formed, along with a viscous brown oil. The brown oil rapidly solidified and formed crystals when the sample was perturbed. The crystals were then analysed using X-ray diffraction, which revealed them to be the intended product (mol­ecular weight: 1847.33 g mol^−1^). The resulting clear crystals were remarkably air-sensitive, decomposing within a few seconds of contact with air after removal from the mother liquor. Weighing of the crystals was not attempted due to their sensitive nature.

^19^F{^1^H} NMR (564 MHz, penta­fluoro­benzene solvent, δ): −76.6 (36 F, *s*, [Al(OC(CF_3_)_3_)_4_]^−^), −132.6 (8 F, *m*, *ortho* C_6_F_5_CN), −141.6 (4 F, *tt*, ^3^*J*_FF_ = 18.8 Hz, ^4^*J*_FF_ = 7.4 Hz, *para* C_6_F_5_CN), −159.9 (8 F, *m*, *meta* C_6_F_5_CN).

The ^19^F NMR spectrum was referenced relative to CFCl_3_ using the IUPAC recommended indirect referencing method (Harris *et al.*, 2001 ▸). This was achieved by setting the 0 ppm frequency of the ^19^F spectrum equal to 0.94094011 times the frequency of 0 ppm in the ^1^H NMR spectrum.

## 6. Refinement

Crystal data, data collection and structure refinement details are summarized in Table 1 ▸. The structure was refined in the chiral space group *P*2_1_ with two crystallographically independent ion pairs in the asymmetric unit (*Z*′ = 2). Refinement converged satisfactorily to *R*1 = 0.051 [for *I* > 2σ(*I*)] and *wR*2 = 0.137 for all data, with a goodness-of-fit of 1.03. The relatively large number of refined parameters (2063) reflects the presence of two independent cations and two independent anions in the asymmetric unit, resulting in a comparatively modest data-to-parameter ratio in the diffraction data.

The absolute structure was refined as an inversion twin, giving a Flack parameter of 0.49 (2). This value indicates that the crystal examined consists of approximately equal proportions of the two inversion-related twin domains and, consequently, the absolute configuration cannot be determined from the diffraction data. The final difference Fourier map showed no chemically significant residual electron density, with residual peaks of +0.84 and −0.54 e Å^−3^, located in the vicinity of the heavy Ag atoms.

The disorder of one of the OC(CF_3_)_3_ groups was modelled over two orientations using the disordered structure refinement (DSR) approach. Appropriate restraints and equivalent-atom constraints were applied to maintain chemically reasonable geometry and displacement parameters during refinement.

## Supplementary Material

Crystal structure: contains datablock(s) I. DOI: 10.1107/S205698902600976X/vu2025sup1.cif

Supporting information file. DOI: 10.1107/S205698902600976X/vu2025Isup4.cdx

Structure factors: contains datablock(s) I. DOI: 10.1107/S205698902600976X/vu2025Isup6.hkl

19F NMR 1D and COSY spectra. DOI: 10.1107/S205698902600976X/vu2025sup3.pdf

Supporting information file. DOI: 10.1107/S205698902600976X/vu2025sup5.mol

CCDC reference: 2588967

Additional supporting information:  crystallographic information; 3D view; checkCIF report

## Figures and Tables

**Figure 1:**
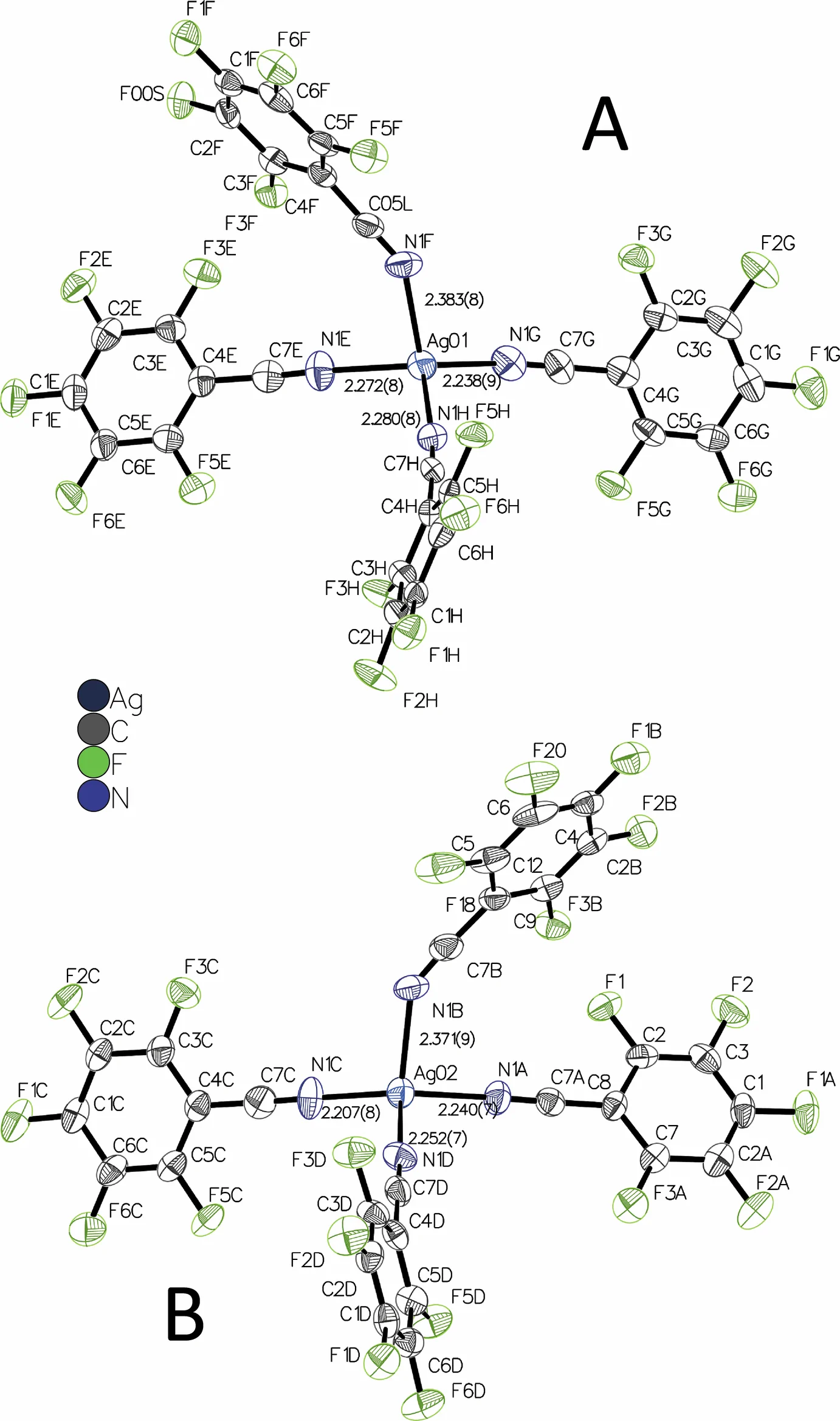
The two crystallographically independent mol­ecules (*a*) *A* and (*b*) *B* in the asymmetric unit, with Ag-coordination bond distances indicated on the figure.

**Figure 2:**
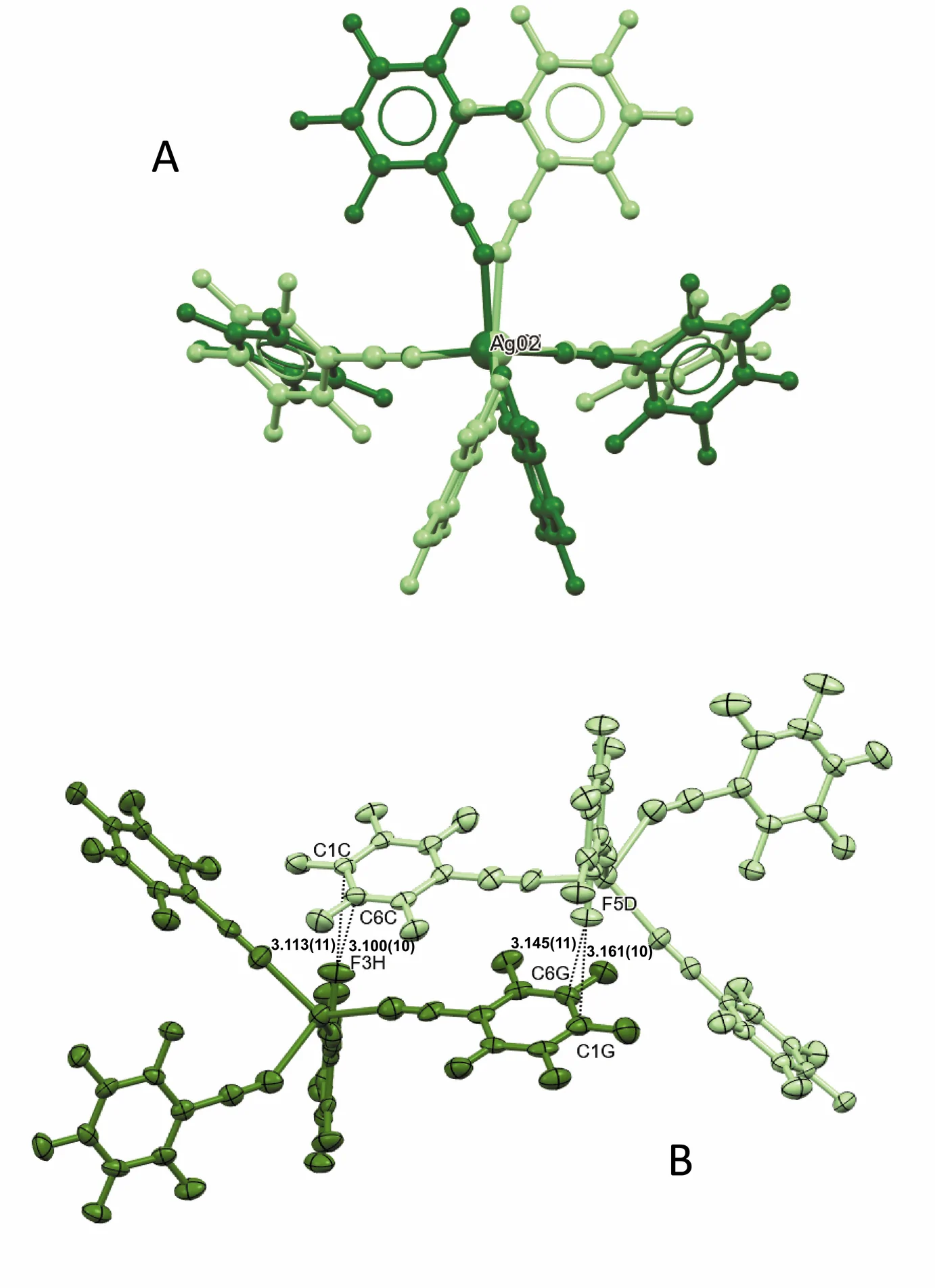
(*a*) Overlay of the two crystallographically independent cations, showing their approximate non-crystallographic mirror relationship. (*b*) The pair is associated by C—F⋯π contacts, generating a pseudo-centre of inversion between them.

**Figure 3:**
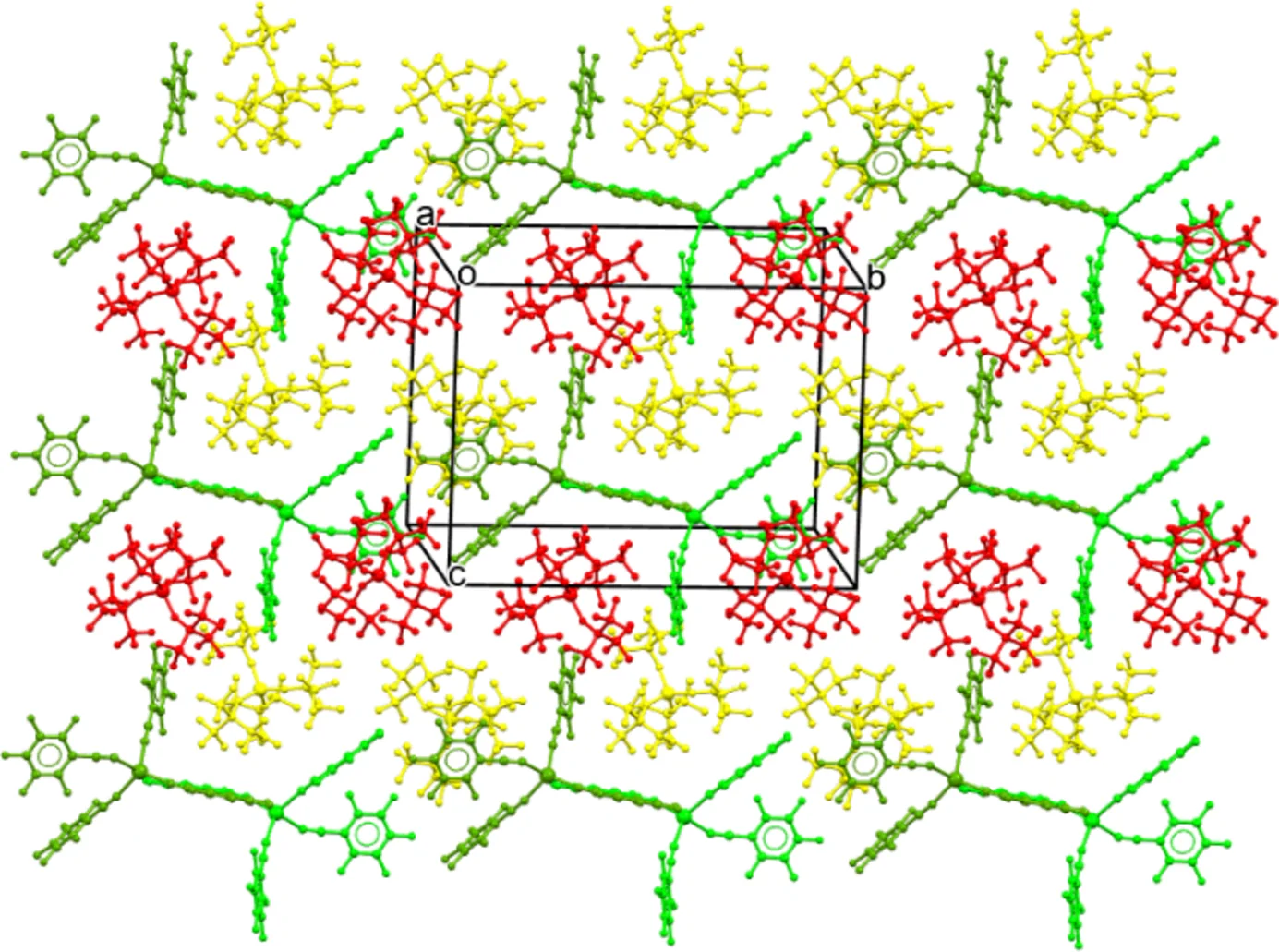
Packing of mol­ecules viewed down an arbitrary direction.

**Table 1:** Experimental details

Crystal data	
Chemical formula	[Ag(C_7_F_5_N)_4_][Al(C_4_F_9_O)_4_]
*M*_r_	1847.33
Crystal system, space group	Monoclinic, *P*2_1_
Temperature (K)	120
*a*, *b*, *c* (Å)	13.3210 (9), 24.5685 (16), 17.8741 (9)
β (°)	98.493 (3)
*V* (Å^3^)	5785.6 (6)
*Z*	4
Radiation type	Mo *K*α
μ (mm^−1^)	0.59
Crystal size (mm)	0.22 × 0.19 × 0.12

Data collection	
Diffractometer	Bruker D8 Quest
Absorption correction	Multi-scan (*SADABS*; Krause *et al.*, 2015 ▸)
*T*_min_, *T*_max_	0.450, 0.745
No. of measured, independent and observed [*I* > 2σ(*I*)] reflections	72495, 24108, 18810
*R*_int_	0.065
(sin θ/λ)_max_ (Å^−1^)	0.634

Refinement	
*R*[*F*^2^ > 2σ(*F*^2^)], *wR*(*F*^2^), *S*	0.051, 0.137, 1.03
No. of reflections	24108
No. of parameters	2063
No. of restraints	80
Δρ_max_, Δρ_min_ (e Å^−3^)	0.84, −0.54
Absolute structure	Refined as an inversion twin.
Absolute structure parameter	0.49 (2)

Computer programs: *APEX6* and *SAINT* (Bruker, 2024 ▸), *SHELXT* (Sheldrick, 2015*a* ▸), *SHELXL* (Sheldrick, 2015*b* ▸) and *OLEX2* (Dolomanov *et al.*, 2009 ▸).

## supplementary crystallographic information

### Tetrakis(perfluorobenzonitrile)silver(I) tetrakis(perfluoro-tert-butoxy)aluminate(1-) Crystal data

**Table:**

[Ag(C_7_F_5_N)_4_][Al(C_4_F_9_O)_4_]	*F*(000) = 3552
*M**_r_* = 1847.33	*D*_x_ = 2.121 Mg m^−^^3^
Monoclinic, *P*2_1_	Mo *K*α radiation, λ = 0.71073 Å
*a* = 13.3210 (9) Å	Cell parameters from 2527 reflections
*b* = 24.5685 (16) Å	θ = 2.6–25.0°
*c* = 17.8741 (9) Å	µ = 0.59 mm^−^^1^
β = 98.493 (3)°	*T* = 120 K
*V* = 5785.6 (6) Å^3^	Plate, colourless
*Z* = 4	0.22 × 0.19 × 0.12 mm

### Tetrakis(perfluorobenzonitrile)silver(I) tetrakis(perfluoro-tert-butoxy)aluminate(1-) Data collection

**Table:**

Bruker D8 Quest four-circle diffractometer	18810 reflections with *I* > 2σ(*I*)
ω and φ scans	*R*_int_ = 0.065
Absorption correction: multi-scan (SADABS; Krause *et al.*, 2015)	θ_max_ = 26.8°, θ_min_ = 2.0°
*T*_min_ = 0.450, *T*_max_ = 0.745	*h* = −16→16
72495 measured reflections	*k* = −31→31
24108 independent reflections	*l* = −22→22

### Tetrakis(perfluorobenzonitrile)silver(I) tetrakis(perfluoro-tert-butoxy)aluminate(1-) Refinement

**Table:**

Refinement on *F*^2^	80 restraints
Least-squares matrix: full	*w* = 1/[σ^2^(*F*_o_^2^) + (0.0681*P*)^2^ + 2.0284*P*] where *P* = (*F*_o_^2^ + 2*F*_c_^2^)/3
*R*[*F*^2^ > 2σ(*F*^2^)] = 0.051	(Δ/σ)_max_ = 0.001
*wR*(*F*^2^) = 0.137	Δρ_max_ = 0.84 e Å^−^^3^
*S* = 1.03	Δρ_min_ = −0.54 e Å^−^^3^
24108 reflections	Absolute structure: Refined as an inversion twin.
2063 parameters	Absolute structure parameter: 0.49 (2)

### Tetrakis(perfluorobenzonitrile)silver(I) tetrakis(perfluoro-tert-butoxy)aluminate(1-) Special details

**Table:**

Geometry. All esds (except the esd in the dihedral angle between two l.s. planes) are estimated using the full covariance matrix. The cell esds are taken into account individually in the estimation of esds in distances, angles and torsion angles; correlations between esds in cell parameters are only used when they are defined by crystal symmetry. An approximate (isotropic) treatment of cell esds is used for estimating esds involving l.s. planes.
Refinement. Refined as a 2-component inversion twin. Single-crystal X-ray diffraction data was collected at 120 K on a Bruker D8 Quest diffractometer equipped with a Photon IV detector and a microfocus Mo Kα radiation source (λ = 0.71073 Å ). Symmetry-related absorption corrections were applied using SADABS program, and the data were corrected for Lorentz and polarization effects using Bruker APEX6 software (Bruker, 2024). The structure was solved by intrinsic phasing with SHELXT (Sheldrick, 2015a) and refined by full-matrix least-squares on F^2^ using SHELXL (Sheldrick, 2015b) within the Olex2 program (Dolomanov *et al.*, 2009).

### Tetrakis(perfluorobenzonitrile)silver(I) tetrakis(perfluoro-tert-butoxy)aluminate(1-) Fractional atomic coordinates and isotropic or equivalent isotropic displacement parameters (Å^2^)

**Table:**

	*x*	*y*	*z*	*U*_iso_*/*U*_eq_	Occ. (<1)
Ag01	−0.26559 (5)	0.12413 (3)	0.18260 (4)	0.04260 (17)	
Ag02	−0.75334 (5)	0.28504 (3)	0.68125 (4)	0.04215 (17)	
Al1A	−0.13251 (16)	−0.18574 (8)	0.94405 (12)	0.0230 (4)	
Al1B	−0.36406 (15)	0.08574 (8)	0.57325 (12)	0.0224 (4)	
F5B	−0.4541 (4)	−0.0479 (2)	0.7599 (3)	0.0468 (12)	
F6B	−0.4883 (4)	−0.03406 (19)	0.6409 (3)	0.0494 (13)	
F7B	−0.3339 (4)	−0.0391 (2)	0.6923 (3)	0.0486 (12)	
F9B	−0.5376 (4)	0.1119 (2)	0.7399 (3)	0.0487 (13)	
F10B	−0.6030 (4)	0.0331 (2)	0.7120 (3)	0.0493 (13)	
F11B	−0.5155 (4)	0.0465 (2)	0.8213 (3)	0.0548 (14)	
F13B	−0.3395 (4)	0.1073 (2)	0.7983 (3)	0.0495 (13)	
F14B	−0.2468 (3)	0.0570 (2)	0.7379 (3)	0.0465 (12)	
F15B	−0.3130 (4)	0.0231 (2)	0.8303 (3)	0.0532 (13)	
F19B	−0.6348 (5)	0.0542 (3)	0.5577 (4)	0.0455 (16)	0.75
F20B	−0.5471 (14)	0.0110 (6)	0.4868 (8)	0.049 (3)	0.75
F21B	−0.6943 (6)	0.0422 (4)	0.4417 (6)	0.041 (2)	0.75
F23B	−0.5895 (6)	0.2014 (3)	0.4669 (7)	0.071 (3)	0.75
F24B	−0.5882 (7)	0.1605 (4)	0.5723 (5)	0.064 (2)	0.75
F25B	−0.7141 (5)	0.1483 (3)	0.4837 (5)	0.054 (2)	0.75
F27B	−0.4922 (6)	0.1466 (4)	0.3655 (4)	0.060 (2)	0.75
F28B	−0.5343 (7)	0.0619 (4)	0.3542 (5)	0.072 (2)	0.75
F29B	−0.6484 (7)	0.1241 (5)	0.3468 (6)	0.057 (3)	0.75
F33B	−0.1319 (5)	−0.0051 (3)	0.6222 (3)	0.0652 (17)	
F34B	−0.0854 (4)	−0.0471 (2)	0.5269 (4)	0.0568 (15)	
F35B	−0.2114 (4)	−0.0763 (2)	0.5768 (3)	0.0535 (14)	
F37B	−0.3427 (6)	0.0001 (3)	0.3860 (3)	0.077 (2)	
F38B	−0.2377 (5)	−0.0664 (2)	0.4139 (3)	0.0590 (15)	
F39B	−0.3711 (4)	−0.0585 (2)	0.4679 (4)	0.0654 (17)	
F41B	−0.1281 (6)	0.0277 (2)	0.4154 (4)	0.076 (2)	
F42B	−0.1080 (4)	0.0714 (2)	0.5206 (4)	0.0609 (16)	
F43B	−0.2380 (5)	0.0876 (2)	0.4377 (3)	0.0636 (17)	
F47B	−0.2405 (5)	0.2714 (2)	0.5346 (3)	0.0591 (15)	
F48B	−0.2721 (5)	0.1975 (2)	0.4718 (3)	0.0597 (15)	
F49B	−0.3874 (5)	0.2311 (2)	0.5274 (4)	0.0664 (17)	
F51B	−0.1006 (5)	0.2411 (3)	0.6513 (4)	0.0734 (19)	
F52B	−0.0917 (5)	0.1884 (3)	0.5565 (4)	0.0672 (18)	
F53B	−0.0909 (5)	0.1549 (3)	0.6673 (4)	0.074 (2)	
F55B	−0.2423 (7)	0.1949 (3)	0.7378 (3)	0.087 (2)	
F56B	−0.3903 (5)	0.1949 (2)	0.6709 (4)	0.0698 (18)	
F57B	−0.2947 (7)	0.2656 (2)	0.6734 (4)	0.079 (2)	
O2B	−0.4249 (4)	0.0685 (2)	0.6485 (3)	0.0264 (11)	
O16B	−0.4548 (4)	0.1083 (2)	0.5013 (3)	0.0337 (12)	
O30B	−0.3058 (4)	0.0277 (2)	0.5465 (3)	0.0301 (11)	
O44B	−0.2738 (4)	0.1349 (2)	0.5934 (3)	0.0321 (12)	
C3B	−0.4238 (5)	0.0415 (3)	0.7144 (4)	0.0274 (15)	
C4B	−0.4258 (6)	−0.0206 (3)	0.7024 (5)	0.0355 (18)	
C8B	−0.5214 (7)	0.0578 (4)	0.7478 (4)	0.0396 (19)	
C12B	−0.3289 (6)	0.0572 (3)	0.7710 (4)	0.0358 (17)	
C17B	−0.5547 (7)	0.1060 (4)	0.4709 (5)	0.0274 (19)	0.75
C18B	−0.6080 (8)	0.0523 (5)	0.4873 (6)	0.036 (2)	0.75
C22B	−0.6108 (9)	0.1553 (5)	0.4971 (9)	0.052 (3)	0.75
C26B	−0.5603 (10)	0.1100 (6)	0.3833 (7)	0.051 (4)	0.75
C31B	−0.2396 (6)	0.0054 (3)	0.5049 (4)	0.0280 (15)	
C32B	−0.1658 (6)	−0.0315 (4)	0.5588 (5)	0.0394 (18)	
C36B	−0.2988 (7)	−0.0314 (4)	0.4436 (5)	0.043 (2)	
C40B	−0.1790 (8)	0.0487 (4)	0.4691 (5)	0.047 (2)	
C45B	−0.2485 (6)	0.1882 (3)	0.6057 (5)	0.0372 (18)	
C46B	−0.2873 (7)	0.2221 (3)	0.5331 (5)	0.044 (2)	
C50B	−0.1324 (8)	0.1934 (4)	0.6207 (7)	0.055 (3)	
C54B	−0.2972 (10)	0.2119 (4)	0.6716 (7)	0.063 (3)	
F00S	−0.3365 (4)	0.4199 (2)	0.2246 (3)	0.0471 (12)	
F1	−0.7154 (4)	0.1388 (2)	0.9143 (3)	0.0518 (13)	
F1A	−0.3989 (5)	0.0780 (2)	1.0332 (3)	0.0580 (16)	
F1B	−0.8705 (4)	−0.0587 (2)	0.6076 (4)	0.0586 (15)	
F1C	−1.1722 (4)	0.5621 (3)	0.7043 (3)	0.0621 (17)	
F1D	−0.3216 (4)	0.3913 (2)	0.3729 (3)	0.0531 (14)	
F1E	0.1121 (5)	0.3321 (2)	0.5223 (3)	0.0590 (15)	
F1F	−0.3683 (4)	0.4696 (2)	0.0886 (3)	0.0524 (14)	
F1G	−0.6851 (5)	−0.1516 (3)	0.2032 (4)	0.0650 (17)	
F1H	0.1901 (4)	0.0359 (2)	−0.1164 (3)	0.0476 (13)	
F2	−0.6035 (5)	0.0724 (2)	1.0153 (3)	0.0597 (15)	
F2A	−0.3050 (4)	0.1518 (3)	0.9556 (3)	0.0550 (14)	
F2B	−0.8193 (4)	−0.0065 (2)	0.7394 (3)	0.0476 (12)	
F2C	−1.2494 (3)	0.4611 (3)	0.6697 (3)	0.0519 (14)	
F2D	−0.4930 (5)	0.3380 (3)	0.3142 (3)	0.0614 (16)	
F2E	−0.0881 (5)	0.3526 (2)	0.4909 (3)	0.0543 (14)	
F2G	−0.7644 (4)	−0.0522 (3)	0.1737 (3)	0.0532 (14)	
F2H	0.2270 (4)	0.0143 (3)	0.0326 (3)	0.0594 (16)	
F3A	−0.4159 (4)	0.2170 (2)	0.8534 (3)	0.0497 (13)	
F3B	−0.8079 (4)	0.1033 (2)	0.7419 (3)	0.0451 (12)	
F3C	−1.1221 (4)	0.3762 (2)	0.6683 (3)	0.0559 (14)	
F3D	−0.6252 (4)	0.3046 (3)	0.4066 (3)	0.0577 (15)	
F3E	−0.2048 (4)	0.2907 (2)	0.3855 (3)	0.0502 (12)	
F3F	−0.3281 (4)	0.3100 (2)	0.2319 (3)	0.0445 (12)	
F3G	−0.6406 (4)	0.0361 (2)	0.1806 (3)	0.0517 (13)	
F3H	0.0825 (4)	0.0367 (3)	0.1224 (3)	0.0580 (16)	
F5A	0.0278 (4)	−0.0908 (2)	0.9608 (3)	0.0441 (11)	
F5C	−0.8428 (4)	0.4918 (3)	0.7406 (3)	0.0501 (13)	
F5D	−0.4090 (4)	0.3748 (2)	0.6164 (3)	0.0527 (14)	
F5E	0.0787 (4)	0.1856 (2)	0.3549 (3)	0.0499 (13)	
F5F	−0.3850 (4)	0.3013 (2)	−0.0328 (3)	0.0530 (14)	
F5G	−0.3575 (4)	−0.0775 (2)	0.2455 (3)	0.0507 (14)	
F5H	−0.1387 (4)	0.0922 (2)	−0.0912 (3)	0.0603 (16)	
F6A	0.1227 (4)	−0.0682 (2)	0.8785 (3)	0.0487 (12)	
F6C	−0.9695 (5)	0.5772 (3)	0.7375 (4)	0.0662 (17)	
F6D	−0.2796 (4)	0.4101 (3)	0.5222 (3)	0.0589 (15)	
F6E	0.1967 (4)	0.2509 (3)	0.4537 (4)	0.0633 (16)	
F6F	−0.3879 (4)	0.4114 (2)	−0.0401 (3)	0.0504 (13)	
F6G	−0.4824 (4)	−0.1653 (2)	0.2387 (3)	0.0582 (15)	
F6H	0.0076 (5)	0.0735 (2)	−0.1796 (3)	0.0573 (15)	
F7A	0.1306 (4)	−0.1498 (2)	0.9238 (3)	0.0576 (15)	
F9A	0.0537 (4)	−0.2101 (3)	0.8055 (4)	0.0679 (17)	
F10A	−0.0348 (5)	−0.1635 (3)	0.7137 (3)	0.0641 (16)	
F11A	0.1162 (4)	−0.1375 (3)	0.7623 (4)	0.0721 (19)	
F13A	−0.1582 (4)	−0.0907 (2)	0.7569 (3)	0.0533 (14)	
F14A	−0.0153 (4)	−0.0499 (3)	0.7557 (4)	0.072 (2)	
F15A	−0.0915 (4)	−0.0410 (2)	0.8525 (4)	0.0577 (14)	
F18	−0.9050 (5)	0.1073 (3)	0.4782 (3)	0.074 (2)	
F19A	−0.4369 (4)	−0.2423 (3)	0.8389 (4)	0.0608 (16)	
F20	−0.9084 (5)	−0.0028 (3)	0.4762 (3)	0.0697 (19)	
F20A	−0.4060 (5)	−0.3295 (3)	0.8332 (4)	0.0697 (18)	
F21A	−0.3819 (5)	−0.2874 (3)	0.9396 (3)	0.0677 (18)	
F23A	−0.2179 (7)	−0.3488 (2)	0.9255 (4)	0.087 (2)	
F24A	−0.2131 (6)	−0.3524 (3)	0.8033 (5)	0.092 (3)	
F25A	−0.1010 (5)	−0.3082 (3)	0.8775 (4)	0.079 (2)	
F27A	−0.2945 (4)	−0.1925 (2)	0.7738 (3)	0.0505 (13)	
F28A	−0.1713 (5)	−0.2464 (3)	0.7554 (3)	0.0737 (19)	
F29A	−0.3285 (6)	−0.2709 (3)	0.7237 (3)	0.077 (2)	
F33A	−0.3336 (4)	−0.1937 (2)	1.0486 (3)	0.0504 (13)	
F34A	−0.2658 (4)	−0.1418 (2)	1.1366 (3)	0.0534 (13)	
F35A	−0.4127 (4)	−0.1215 (2)	1.0746 (3)	0.0478 (13)	
F37A	−0.4020 (3)	−0.1440 (2)	0.9196 (3)	0.0424 (11)	
F38A	−0.4052 (4)	−0.0579 (2)	0.9452 (3)	0.0436 (12)	
F39A	−0.2935 (3)	−0.0900 (2)	0.8810 (2)	0.0398 (11)	
F41A	−0.1472 (4)	−0.0594 (2)	1.0964 (3)	0.0507 (13)	
F42A	−0.2080 (4)	−0.02112 (19)	0.9920 (3)	0.0550 (14)	
F43A	−0.2996 (4)	−0.0297 (2)	1.0818 (3)	0.0499 (13)	
F47A	0.0999 (5)	−0.2015 (3)	1.1881 (3)	0.0623 (16)	
F48A	−0.0440 (6)	−0.1656 (3)	1.1504 (4)	0.090 (2)	
F49A	0.0828 (8)	−0.1470 (3)	1.0963 (4)	0.103 (3)	
F51A	0.1805 (5)	−0.2329 (3)	1.0475 (5)	0.090 (3)	
F52A	0.1463 (6)	−0.2936 (3)	1.1186 (4)	0.098 (3)	
F53A	0.0882 (4)	−0.3004 (3)	1.0030 (3)	0.0626 (17)	
F55A	−0.0775 (5)	−0.3175 (3)	1.0632 (4)	0.0771 (19)	
F56A	−0.0357 (6)	−0.2858 (3)	1.1713 (4)	0.092 (2)	
F57A	−0.1582 (5)	−0.2507 (3)	1.0928 (4)	0.080 (2)	
O2A	−0.0884 (4)	−0.1586 (2)	0.8661 (3)	0.0293 (11)	
O16A	−0.2277 (4)	−0.2317 (2)	0.9105 (3)	0.0302 (11)	
O30A	−0.1834 (3)	−0.1372 (2)	0.9973 (3)	0.0272 (10)	
O44A	−0.0296 (4)	−0.2136 (2)	1.0004 (3)	0.0311 (11)	
N1A	−0.6743 (6)	0.2383 (3)	0.7804 (4)	0.0428 (17)	
N1B	−0.8452 (6)	0.2119 (4)	0.6173 (5)	0.055 (2)	
N1C	−0.8581 (6)	0.3516 (4)	0.6998 (4)	0.051 (2)	
N1D	−0.6432 (6)	0.3068 (3)	0.6018 (4)	0.0426 (17)	
N1E	−0.1771 (7)	0.1763 (4)	0.2746 (5)	0.053 (2)	
N1F	−0.3440 (6)	0.1996 (3)	0.1132 (5)	0.0477 (19)	
N1G	−0.3776 (6)	0.0626 (4)	0.2117 (4)	0.0500 (19)	
N1H	−0.1594 (6)	0.0907 (3)	0.1047 (5)	0.0440 (18)	
C1	−0.4543 (7)	0.1117 (3)	0.9841 (5)	0.039 (2)	
C1C	−1.1108 (6)	0.5195 (4)	0.7032 (5)	0.039 (2)	
C1D	−0.3848 (7)	0.3735 (4)	0.4179 (5)	0.0375 (19)	
C1E	0.0550 (7)	0.3015 (4)	0.4732 (5)	0.044 (2)	
C1F	−0.3635 (6)	0.4161 (4)	0.0917 (5)	0.0365 (19)	
C1G	−0.6243 (7)	−0.1087 (4)	0.2063 (5)	0.045 (2)	
C1H	0.1192 (6)	0.0451 (3)	−0.0730 (5)	0.0336 (18)	
C2	−0.6138 (6)	0.1415 (3)	0.9241 (5)	0.0359 (18)	
C2A	−0.4061 (7)	0.1482 (4)	0.9441 (5)	0.041 (2)	
C2B	−0.8394 (6)	0.0220 (4)	0.6760 (5)	0.0350 (18)	
C2C	−1.1504 (6)	0.4684 (4)	0.6848 (4)	0.0343 (18)	
C2D	−0.4712 (7)	0.3466 (4)	0.3891 (5)	0.0377 (19)	
C2E	−0.0473 (7)	0.3126 (3)	0.4557 (5)	0.041 (2)	
C2F	−0.3495 (6)	0.3904 (4)	0.1625 (5)	0.0352 (18)	
C2G	−0.6645 (7)	−0.0579 (5)	0.1931 (5)	0.044 (2)	
C2H	0.1368 (6)	0.0352 (4)	0.0039 (5)	0.0371 (18)	
C3	−0.5586 (7)	0.1081 (3)	0.9741 (5)	0.0384 (19)	
C3A	−0.0181 (5)	−0.1273 (3)	0.8380 (4)	0.0298 (16)	
C3C	−1.0836 (7)	0.4255 (4)	0.6857 (5)	0.040 (2)	
C3D	−0.5388 (7)	0.3291 (4)	0.4363 (5)	0.040 (2)	
C3E	−0.1074 (6)	0.2805 (4)	0.4040 (4)	0.0372 (18)	
C3F	−0.3445 (6)	0.3343 (4)	0.1655 (4)	0.0328 (17)	
C3G	−0.6011 (6)	−0.0128 (4)	0.1939 (4)	0.0367 (19)	
C3H	0.0655 (6)	0.0465 (3)	0.0480 (5)	0.0349 (18)	
C4	−0.8653 (6)	−0.0054 (4)	0.6081 (5)	0.0384 (19)	
C4A	0.0675 (6)	−0.1077 (4)	0.9013 (5)	0.0404 (19)	
C4C	−0.9802 (6)	0.4320 (4)	0.7032 (4)	0.0349 (18)	
C4D	−0.5178 (6)	0.3375 (3)	0.5137 (5)	0.0326 (17)	
C4E	−0.0654 (6)	0.2364 (3)	0.3706 (5)	0.0345 (18)	
C4F	−0.3584 (6)	0.3038 (4)	0.0993 (5)	0.0335 (18)	
C4G	−0.4969 (6)	−0.0192 (4)	0.2133 (4)	0.039 (2)	
C4H	−0.0299 (6)	0.0658 (3)	0.0175 (5)	0.0311 (17)	
C5	−0.8820 (7)	0.0793 (5)	0.5426 (5)	0.050 (2)	
C5C	−0.9432 (6)	0.4844 (4)	0.7219 (4)	0.0360 (18)	
C5D	−0.4292 (7)	0.3656 (4)	0.5426 (5)	0.0381 (19)	
C5E	0.0379 (7)	0.2266 (4)	0.3880 (5)	0.0381 (19)	
C5F	−0.3719 (6)	0.3295 (4)	0.0299 (5)	0.0386 (19)	
C5G	−0.4574 (6)	−0.0706 (4)	0.2280 (4)	0.0363 (19)	
C5H	−0.0492 (6)	0.0746 (3)	−0.0615 (5)	0.0342 (18)	
C05L	−0.3489 (6)	0.2454 (4)	0.1050 (5)	0.0388 (19)	
C6	−0.8854 (7)	0.0243 (5)	0.5411 (5)	0.050 (3)	
C6C	−1.0073 (6)	0.5270 (4)	0.7221 (5)	0.041 (2)	
C6D	−0.3626 (7)	0.3828 (4)	0.4952 (5)	0.0396 (19)	
C6E	0.0986 (7)	0.2587 (4)	0.4398 (5)	0.044 (2)	
C6F	−0.3749 (6)	0.3862 (4)	0.0271 (5)	0.040 (2)	
C6G	−0.5198 (6)	−0.1157 (4)	0.2240 (5)	0.0388 (19)	
C6H	0.0228 (8)	0.0653 (3)	−0.1058 (5)	0.042 (2)	
C7	−0.4622 (6)	0.1820 (3)	0.8941 (4)	0.0313 (17)	
C7A	−0.6256 (6)	0.2120 (3)	0.8257 (5)	0.0360 (18)	
C7B	−0.8529 (7)	0.1664 (4)	0.6138 (5)	0.045 (2)	
C7C	−0.9124 (7)	0.3881 (4)	0.7022 (5)	0.044 (2)	
C7D	−0.5876 (7)	0.3202 (3)	0.5621 (5)	0.0381 (19)	
C7E	−0.1271 (7)	0.2020 (4)	0.3173 (5)	0.0398 (19)	
C7G	−0.4304 (6)	0.0267 (4)	0.2124 (4)	0.040 (2)	
C7H	−0.1042 (6)	0.0791 (3)	0.0645 (5)	0.0343 (18)	
C8	−0.5681 (6)	0.1794 (3)	0.8807 (5)	0.0330 (17)	
C8A	0.0309 (7)	−0.1584 (4)	0.7799 (6)	0.049 (2)	
C9	−0.8557 (6)	0.1075 (4)	0.6099 (5)	0.0373 (19)	
C12	−0.8352 (6)	0.0775 (4)	0.6769 (5)	0.0354 (18)	
C12A	−0.0714 (7)	−0.0756 (4)	0.8020 (6)	0.048 (2)	
C17A	−0.2626 (7)	−0.2681 (3)	0.8566 (5)	0.0370 (18)	
C18A	−0.3721 (9)	−0.2821 (4)	0.8657 (6)	0.053 (3)	
C22A	−0.1972 (10)	−0.3193 (5)	0.8641 (7)	0.071 (4)	
C26A	−0.2634 (8)	−0.2435 (4)	0.7765 (5)	0.051 (2)	
C31A	−0.2666 (5)	−0.1099 (3)	1.0117 (4)	0.0264 (15)	
C32A	−0.3224 (7)	−0.1426 (4)	1.0675 (5)	0.042 (2)	
C36A	−0.3427 (6)	−0.1004 (3)	0.9381 (4)	0.0332 (16)	
C40A	−0.2321 (6)	−0.0551 (3)	1.0471 (5)	0.041 (2)	
C45A	0.0128 (6)	−0.2327 (3)	1.0687 (4)	0.0340 (17)	
C46A	0.0411 (9)	−0.1855 (5)	1.1262 (6)	0.057 (3)	
C50A	0.1083 (7)	−0.2659 (4)	1.0567 (6)	0.048 (2)	
C54A	−0.0620 (8)	−0.2706 (4)	1.1020 (6)	0.053 (3)	
C17C	−0.5440 (16)	0.1093 (10)	0.4584 (13)	0.0274 (19)	0.25
C26C	−0.534 (2)	0.1336 (14)	0.3806 (15)	0.051 (4)	0.25
F29C	−0.610 (2)	0.121 (2)	0.3277 (17)	0.089 (15)	0.25
F27C	−0.529 (2)	0.1884 (10)	0.3845 (16)	0.076 (7)	0.25
F28C	−0.448 (2)	0.1153 (12)	0.3547 (13)	0.070 (7)	0.25
F21C	−0.684 (2)	0.0539 (15)	0.427 (2)	0.041 (2)	0.25
C18C	−0.583 (2)	0.0504 (10)	0.4473 (16)	0.036 (2)	0.25
F20C	−0.546 (2)	0.0251 (11)	0.3936 (14)	0.075 (8)	0.25
F19C	−0.560 (4)	0.0207 (19)	0.511 (2)	0.068 (15)	0.25
C22C	−0.617 (2)	0.1428 (13)	0.5000 (17)	0.052 (3)	0.25
F23C	−0.5748 (17)	0.1878 (10)	0.529 (2)	0.069 (8)	0.25
F25C	−0.6984 (17)	0.1573 (11)	0.4474 (15)	0.054 (2)	0.25
F24C	−0.6372 (18)	0.1117 (12)	0.5588 (15)	0.097 (12)	0.25

### Tetrakis(perfluorobenzonitrile)silver(I) tetrakis(perfluoro-tert-butoxy)aluminate(1-) Atomic displacement parameters (Å^2^)

**Table:**

	*U*^11^	*U*^22^	*U*^33^	*U*^12^	*U*^13^	*U*^23^
Ag01	0.0375 (3)	0.0429 (4)	0.0458 (4)	0.0009 (3)	0.0009 (3)	−0.0071 (3)
Ag02	0.0384 (3)	0.0410 (4)	0.0459 (4)	0.0023 (3)	0.0022 (3)	0.0093 (3)
Al1A	0.0256 (10)	0.0221 (10)	0.0206 (10)	0.0039 (8)	0.0010 (8)	−0.0014 (8)
Al1B	0.0244 (10)	0.0207 (10)	0.0205 (10)	0.0009 (8)	−0.0024 (8)	−0.0010 (8)
F5B	0.054 (3)	0.035 (3)	0.051 (3)	−0.003 (2)	0.005 (2)	0.011 (2)
F6B	0.064 (3)	0.032 (2)	0.045 (3)	−0.001 (2)	−0.016 (2)	−0.003 (2)
F7B	0.053 (3)	0.040 (3)	0.053 (3)	0.016 (2)	0.010 (2)	0.006 (2)
F9B	0.054 (3)	0.043 (3)	0.054 (3)	0.012 (2)	0.023 (2)	−0.009 (2)
F10B	0.030 (2)	0.056 (3)	0.061 (3)	−0.003 (2)	0.003 (2)	0.003 (3)
F11B	0.065 (3)	0.069 (4)	0.037 (3)	−0.003 (3)	0.028 (2)	0.003 (3)
F13B	0.061 (3)	0.051 (3)	0.034 (3)	−0.013 (2)	0.000 (2)	−0.008 (2)
F14B	0.027 (2)	0.067 (3)	0.042 (3)	−0.001 (2)	−0.004 (2)	0.006 (2)
F15B	0.063 (3)	0.061 (3)	0.030 (2)	−0.013 (3)	−0.012 (2)	0.017 (2)
F19B	0.043 (4)	0.059 (4)	0.034 (3)	−0.009 (3)	0.003 (3)	0.007 (3)
F20B	0.041 (5)	0.036 (5)	0.070 (7)	0.009 (4)	0.009 (5)	0.011 (5)
F21B	0.045 (3)	0.035 (5)	0.039 (5)	−0.013 (3)	−0.012 (3)	0.014 (3)
F23B	0.051 (5)	0.030 (4)	0.131 (9)	0.006 (3)	0.005 (5)	0.018 (5)
F24B	0.064 (5)	0.057 (5)	0.071 (6)	0.004 (4)	0.013 (4)	−0.028 (5)
F25B	0.031 (3)	0.043 (4)	0.090 (7)	0.005 (3)	0.017 (4)	0.013 (4)
F27B	0.044 (4)	0.093 (7)	0.039 (4)	−0.026 (4)	−0.008 (3)	0.028 (4)
F28B	0.090 (6)	0.091 (7)	0.036 (4)	0.000 (5)	0.019 (4)	−0.017 (5)
F29B	0.040 (5)	0.078 (6)	0.046 (6)	−0.012 (5)	−0.021 (4)	0.034 (5)
F33B	0.065 (4)	0.077 (4)	0.045 (3)	0.022 (3)	−0.022 (3)	−0.010 (3)
F34B	0.040 (3)	0.053 (3)	0.080 (4)	0.018 (2)	0.018 (3)	−0.003 (3)
F35B	0.061 (3)	0.039 (3)	0.066 (3)	0.016 (2)	0.024 (3)	0.018 (3)
F37B	0.105 (5)	0.073 (4)	0.042 (3)	0.037 (4)	−0.027 (3)	−0.019 (3)
F38B	0.074 (4)	0.047 (3)	0.057 (3)	0.016 (3)	0.011 (3)	−0.024 (3)
F39B	0.049 (3)	0.046 (3)	0.100 (5)	−0.010 (3)	0.007 (3)	−0.031 (3)
F41B	0.111 (5)	0.050 (3)	0.087 (5)	0.010 (3)	0.078 (4)	−0.002 (3)
F42B	0.050 (3)	0.046 (3)	0.094 (5)	−0.015 (2)	0.032 (3)	−0.018 (3)
F43B	0.105 (5)	0.036 (3)	0.058 (4)	0.015 (3)	0.038 (3)	0.016 (3)
F47B	0.092 (4)	0.033 (3)	0.055 (3)	−0.014 (3)	0.019 (3)	0.000 (2)
F48B	0.099 (5)	0.040 (3)	0.037 (3)	−0.004 (3)	−0.002 (3)	0.004 (2)
F49B	0.057 (3)	0.040 (3)	0.097 (5)	0.013 (2)	−0.005 (3)	0.006 (3)
F51B	0.063 (4)	0.059 (4)	0.092 (5)	−0.029 (3)	−0.009 (3)	−0.022 (4)
F52B	0.051 (3)	0.060 (4)	0.095 (5)	−0.010 (3)	0.027 (3)	−0.005 (3)
F53B	0.056 (4)	0.070 (4)	0.084 (5)	−0.011 (3)	−0.025 (3)	0.022 (4)
F55B	0.139 (7)	0.083 (5)	0.037 (3)	−0.051 (5)	0.011 (4)	−0.012 (3)
F56B	0.077 (4)	0.050 (3)	0.093 (5)	−0.013 (3)	0.046 (4)	−0.023 (3)
F57B	0.138 (6)	0.037 (3)	0.072 (4)	−0.020 (3)	0.049 (4)	−0.027 (3)
O2B	0.030 (2)	0.028 (3)	0.022 (2)	0.009 (2)	0.004 (2)	0.002 (2)
O16B	0.029 (3)	0.042 (3)	0.027 (3)	0.002 (2)	−0.004 (2)	0.009 (2)
O30B	0.029 (3)	0.030 (3)	0.034 (3)	0.002 (2)	0.012 (2)	−0.005 (2)
O44B	0.036 (3)	0.031 (3)	0.029 (3)	−0.006 (2)	0.003 (2)	−0.006 (2)
C3B	0.031 (4)	0.032 (4)	0.020 (3)	0.003 (3)	0.006 (3)	0.001 (3)
C4B	0.033 (4)	0.037 (4)	0.032 (4)	0.001 (3)	−0.007 (3)	0.008 (3)
C8B	0.050 (5)	0.045 (5)	0.024 (4)	−0.001 (4)	0.007 (3)	0.001 (4)
C12B	0.037 (4)	0.042 (4)	0.025 (4)	−0.001 (3)	−0.006 (3)	0.003 (3)
C17B	0.026 (4)	0.036 (4)	0.018 (4)	0.004 (3)	−0.002 (3)	0.008 (3)
C18B	0.032 (5)	0.041 (5)	0.030 (6)	−0.005 (4)	−0.013 (4)	0.008 (5)
C22B	0.037 (5)	0.039 (8)	0.075 (8)	0.001 (5)	−0.001 (5)	−0.011 (6)
C26B	0.037 (7)	0.075 (11)	0.036 (6)	−0.004 (6)	−0.006 (5)	0.011 (7)
C31B	0.032 (4)	0.027 (4)	0.026 (4)	0.007 (3)	0.007 (3)	−0.004 (3)
C32B	0.039 (4)	0.043 (5)	0.036 (4)	0.008 (4)	0.004 (3)	−0.001 (4)
C36B	0.051 (5)	0.034 (4)	0.044 (5)	0.013 (4)	0.004 (4)	−0.015 (4)
C40B	0.058 (6)	0.040 (5)	0.053 (6)	0.010 (4)	0.034 (5)	−0.005 (4)
C45B	0.047 (5)	0.028 (4)	0.035 (4)	−0.008 (3)	0.001 (4)	−0.005 (3)
C46B	0.060 (6)	0.026 (4)	0.047 (5)	0.001 (4)	0.007 (4)	0.004 (4)
C50B	0.055 (6)	0.041 (5)	0.064 (7)	−0.012 (4)	−0.011 (5)	−0.007 (5)
C54B	0.088 (8)	0.039 (5)	0.069 (8)	−0.020 (5)	0.035 (6)	−0.016 (5)
F00S	0.055 (3)	0.045 (3)	0.040 (3)	−0.010 (2)	0.005 (2)	−0.009 (2)
F1	0.032 (2)	0.060 (3)	0.062 (3)	−0.007 (2)	0.000 (2)	0.003 (3)
F1A	0.078 (4)	0.052 (3)	0.037 (3)	0.024 (3)	−0.015 (3)	0.000 (2)
F1B	0.049 (3)	0.052 (3)	0.077 (4)	−0.002 (3)	0.017 (3)	−0.027 (3)
F1C	0.049 (3)	0.078 (4)	0.053 (3)	0.028 (3)	−0.015 (3)	−0.024 (3)
F1D	0.048 (3)	0.073 (4)	0.041 (3)	−0.002 (3)	0.014 (2)	0.011 (3)
F1E	0.072 (4)	0.039 (3)	0.057 (3)	−0.013 (3)	−0.021 (3)	0.002 (3)
F1F	0.048 (3)	0.042 (3)	0.067 (4)	−0.012 (2)	0.011 (3)	0.015 (3)
F1G	0.060 (4)	0.072 (4)	0.061 (4)	−0.021 (3)	0.003 (3)	0.019 (3)
F1H	0.059 (3)	0.045 (3)	0.044 (3)	0.000 (2)	0.022 (2)	−0.010 (2)
F2	0.075 (4)	0.047 (3)	0.057 (4)	−0.015 (3)	0.008 (3)	0.012 (3)
F2A	0.034 (3)	0.084 (4)	0.045 (3)	0.006 (3)	0.000 (2)	−0.005 (3)
F2B	0.054 (3)	0.043 (3)	0.046 (3)	0.002 (2)	0.005 (2)	0.001 (2)
F2C	0.028 (2)	0.086 (4)	0.039 (3)	0.000 (2)	−0.003 (2)	0.006 (3)
F2D	0.073 (4)	0.083 (4)	0.026 (3)	−0.007 (3)	0.003 (3)	−0.002 (3)
F2E	0.077 (4)	0.034 (3)	0.050 (3)	0.006 (3)	0.003 (3)	0.001 (2)
F2G	0.031 (3)	0.088 (4)	0.040 (3)	−0.001 (2)	0.004 (2)	0.002 (3)
F2H	0.029 (2)	0.099 (5)	0.050 (3)	0.016 (3)	0.006 (2)	0.000 (3)
F3A	0.050 (3)	0.062 (3)	0.039 (3)	−0.002 (3)	0.012 (2)	0.004 (3)
F3B	0.054 (3)	0.047 (3)	0.033 (3)	−0.011 (2)	0.004 (2)	−0.005 (2)
F3C	0.059 (3)	0.048 (3)	0.058 (3)	−0.012 (3)	−0.002 (3)	0.015 (3)
F3D	0.061 (3)	0.070 (4)	0.041 (3)	−0.021 (3)	0.003 (2)	−0.006 (3)
F3E	0.043 (3)	0.058 (3)	0.048 (3)	0.008 (2)	0.003 (2)	0.010 (3)
F3F	0.050 (3)	0.050 (3)	0.031 (3)	−0.001 (2)	−0.001 (2)	0.009 (2)
F3G	0.050 (3)	0.055 (3)	0.046 (3)	0.014 (2)	−0.006 (2)	−0.002 (3)
F3H	0.041 (3)	0.096 (5)	0.037 (3)	0.025 (3)	0.008 (2)	0.012 (3)
F5A	0.050 (3)	0.048 (3)	0.036 (3)	−0.011 (2)	0.012 (2)	−0.010 (2)
F5C	0.027 (2)	0.070 (4)	0.050 (3)	0.000 (2)	−0.004 (2)	0.003 (3)
F5D	0.054 (3)	0.074 (4)	0.029 (3)	−0.011 (3)	0.003 (2)	−0.009 (3)
F5E	0.062 (3)	0.043 (3)	0.045 (3)	0.009 (2)	0.007 (3)	0.000 (2)
F5F	0.057 (3)	0.069 (4)	0.032 (3)	0.013 (3)	0.005 (2)	−0.013 (2)
F5G	0.030 (2)	0.073 (4)	0.048 (3)	0.012 (2)	0.000 (2)	−0.013 (3)
F5H	0.060 (3)	0.060 (3)	0.053 (3)	0.028 (3)	−0.021 (3)	−0.013 (3)
F6A	0.037 (3)	0.051 (3)	0.061 (3)	−0.009 (2)	0.017 (2)	0.001 (3)
F6C	0.066 (4)	0.054 (4)	0.071 (4)	−0.003 (3)	−0.017 (3)	−0.017 (3)
F6D	0.045 (3)	0.084 (4)	0.046 (3)	−0.019 (3)	0.001 (2)	−0.008 (3)
F6E	0.043 (3)	0.057 (3)	0.082 (4)	−0.005 (3)	−0.020 (3)	0.006 (3)
F6F	0.046 (3)	0.069 (4)	0.037 (3)	0.001 (3)	0.008 (2)	0.020 (3)
F6G	0.060 (3)	0.054 (3)	0.057 (4)	0.009 (3)	−0.004 (3)	0.012 (3)
F6H	0.078 (4)	0.061 (4)	0.030 (3)	0.015 (3)	0.000 (3)	0.000 (3)
F7A	0.032 (3)	0.065 (4)	0.072 (4)	0.006 (2)	−0.004 (3)	0.018 (3)
F9A	0.055 (3)	0.071 (4)	0.080 (4)	0.022 (3)	0.015 (3)	−0.024 (4)
F10A	0.071 (4)	0.091 (4)	0.033 (3)	−0.002 (3)	0.016 (3)	−0.022 (3)
F11A	0.043 (3)	0.113 (5)	0.067 (4)	−0.013 (3)	0.031 (3)	−0.028 (4)
F13A	0.028 (2)	0.073 (4)	0.056 (3)	0.001 (2)	−0.002 (2)	0.023 (3)
F14A	0.048 (3)	0.093 (5)	0.076 (4)	−0.012 (3)	0.011 (3)	0.048 (4)
F15A	0.057 (3)	0.035 (3)	0.082 (4)	0.000 (2)	0.012 (3)	0.001 (3)
F18	0.073 (4)	0.107 (6)	0.040 (3)	−0.046 (4)	0.003 (3)	0.013 (3)
F19A	0.037 (3)	0.064 (4)	0.077 (4)	−0.008 (3)	−0.003 (3)	−0.013 (3)
F20	0.068 (4)	0.107 (5)	0.037 (3)	−0.035 (4)	0.019 (3)	−0.028 (3)
F20A	0.078 (4)	0.050 (3)	0.078 (4)	−0.033 (3)	−0.002 (3)	−0.022 (3)
F21A	0.078 (4)	0.076 (4)	0.048 (3)	−0.042 (3)	0.008 (3)	0.009 (3)
F23A	0.139 (7)	0.034 (3)	0.075 (5)	0.008 (4)	−0.029 (4)	0.010 (3)
F24A	0.098 (5)	0.061 (4)	0.105 (6)	0.022 (4)	−0.025 (4)	−0.059 (4)
F25A	0.065 (4)	0.073 (4)	0.090 (5)	0.029 (3)	−0.019 (4)	−0.038 (4)
F27A	0.068 (3)	0.038 (3)	0.039 (3)	−0.013 (2)	−0.011 (2)	0.006 (2)
F28A	0.075 (4)	0.097 (5)	0.053 (4)	−0.022 (4)	0.024 (3)	−0.034 (3)
F29A	0.120 (6)	0.065 (4)	0.036 (3)	−0.035 (4)	−0.021 (3)	−0.003 (3)
F33A	0.067 (3)	0.039 (3)	0.051 (3)	−0.005 (2)	0.025 (3)	0.009 (2)
F34A	0.062 (3)	0.067 (4)	0.031 (3)	0.016 (3)	0.007 (2)	0.007 (2)
F35A	0.037 (3)	0.069 (4)	0.042 (3)	0.006 (2)	0.021 (2)	0.005 (2)
F37A	0.030 (2)	0.043 (3)	0.054 (3)	−0.0035 (19)	0.001 (2)	−0.010 (2)
F38A	0.037 (3)	0.048 (3)	0.046 (3)	0.022 (2)	0.007 (2)	0.002 (2)
F39A	0.034 (2)	0.055 (3)	0.030 (2)	0.003 (2)	0.0052 (19)	0.010 (2)
F41A	0.047 (3)	0.047 (3)	0.053 (3)	0.010 (2)	−0.010 (2)	−0.022 (3)
F42A	0.066 (4)	0.029 (2)	0.073 (4)	−0.013 (2)	0.020 (3)	−0.002 (2)
F43A	0.055 (3)	0.038 (3)	0.059 (3)	0.015 (2)	0.018 (3)	−0.015 (2)
F47A	0.067 (4)	0.078 (4)	0.035 (3)	0.016 (3)	−0.016 (2)	−0.007 (3)
F48A	0.102 (5)	0.097 (5)	0.061 (4)	0.048 (4)	−0.021 (4)	−0.036 (4)
F49A	0.182 (8)	0.056 (4)	0.057 (4)	−0.045 (5)	−0.031 (4)	0.003 (3)
F51A	0.036 (3)	0.080 (5)	0.155 (8)	0.009 (3)	0.011 (4)	0.023 (5)
F52A	0.108 (6)	0.111 (6)	0.060 (4)	0.082 (5)	−0.031 (4)	−0.004 (4)
F53A	0.055 (3)	0.073 (4)	0.056 (3)	0.033 (3)	−0.006 (3)	−0.020 (3)
F55A	0.076 (4)	0.066 (4)	0.091 (5)	−0.022 (3)	0.018 (4)	−0.003 (4)
F56A	0.116 (6)	0.087 (5)	0.064 (4)	−0.009 (4)	−0.013 (4)	0.043 (4)
F57A	0.065 (4)	0.083 (5)	0.097 (5)	0.015 (3)	0.028 (4)	0.041 (4)
O2A	0.028 (3)	0.034 (3)	0.024 (3)	0.002 (2)	−0.002 (2)	−0.004 (2)
O16A	0.036 (3)	0.024 (3)	0.028 (3)	−0.002 (2)	−0.002 (2)	−0.004 (2)
O30A	0.023 (2)	0.029 (3)	0.029 (3)	0.0030 (19)	0.0047 (19)	−0.007 (2)
O44A	0.033 (3)	0.035 (3)	0.023 (2)	0.014 (2)	−0.0038 (19)	−0.004 (2)
N1A	0.044 (4)	0.044 (4)	0.038 (4)	0.008 (3)	−0.003 (3)	0.003 (3)
N1B	0.039 (4)	0.055 (6)	0.067 (6)	−0.019 (4)	−0.007 (4)	0.011 (5)
N1C	0.057 (5)	0.058 (5)	0.037 (4)	0.022 (4)	0.001 (4)	0.012 (4)
N1D	0.050 (4)	0.040 (4)	0.039 (4)	−0.009 (3)	0.011 (3)	0.003 (3)
N1E	0.058 (5)	0.054 (5)	0.044 (5)	−0.014 (4)	0.001 (4)	−0.006 (4)
N1F	0.051 (5)	0.049 (5)	0.042 (4)	0.021 (4)	0.003 (3)	−0.005 (4)
N1G	0.047 (4)	0.062 (5)	0.042 (4)	0.000 (4)	0.007 (3)	−0.002 (4)
N1H	0.036 (4)	0.035 (4)	0.060 (5)	0.002 (3)	0.005 (4)	−0.001 (3)
C1	0.050 (5)	0.037 (5)	0.028 (4)	0.014 (4)	−0.007 (3)	−0.004 (3)
C1C	0.036 (4)	0.053 (5)	0.026 (4)	0.016 (4)	0.001 (3)	−0.009 (4)
C1D	0.047 (5)	0.039 (5)	0.028 (4)	0.009 (4)	0.009 (4)	0.008 (3)
C1E	0.058 (5)	0.035 (5)	0.034 (4)	−0.016 (4)	−0.012 (4)	0.009 (4)
C1F	0.024 (4)	0.040 (5)	0.046 (5)	−0.002 (3)	0.007 (3)	0.013 (4)
C1G	0.039 (5)	0.069 (7)	0.027 (4)	−0.015 (4)	0.004 (4)	0.006 (4)
C1H	0.030 (4)	0.030 (4)	0.042 (5)	−0.003 (3)	0.010 (3)	−0.011 (3)
C2	0.030 (4)	0.036 (4)	0.042 (5)	−0.007 (3)	0.008 (3)	−0.010 (4)
C2A	0.038 (4)	0.052 (5)	0.034 (5)	0.008 (4)	0.002 (4)	−0.010 (4)
C2B	0.025 (4)	0.046 (5)	0.033 (4)	−0.002 (3)	0.003 (3)	−0.008 (4)
C2C	0.031 (4)	0.052 (5)	0.020 (4)	0.006 (3)	0.003 (3)	0.005 (3)
C2D	0.040 (4)	0.043 (5)	0.028 (4)	0.004 (4)	−0.001 (3)	0.000 (4)
C2E	0.061 (5)	0.030 (4)	0.032 (4)	−0.003 (4)	0.009 (4)	0.008 (4)
C2F	0.027 (4)	0.045 (5)	0.031 (4)	−0.004 (3)	−0.001 (3)	−0.009 (4)
C2G	0.035 (4)	0.078 (7)	0.019 (4)	0.005 (4)	0.004 (3)	0.004 (4)
C2H	0.029 (4)	0.046 (5)	0.035 (4)	−0.002 (3)	0.002 (3)	−0.004 (4)
C3	0.057 (5)	0.029 (4)	0.029 (4)	−0.004 (3)	0.008 (4)	−0.006 (3)
C3A	0.018 (3)	0.048 (5)	0.024 (4)	0.001 (3)	0.004 (3)	0.002 (3)
C3C	0.041 (5)	0.046 (5)	0.030 (4)	0.000 (4)	−0.003 (3)	0.009 (4)
C3D	0.053 (5)	0.034 (4)	0.033 (4)	−0.008 (4)	0.002 (4)	−0.003 (4)
C3E	0.039 (4)	0.040 (4)	0.030 (4)	0.002 (4)	−0.002 (3)	0.007 (4)
C3F	0.028 (4)	0.041 (4)	0.030 (4)	−0.001 (3)	0.006 (3)	0.009 (3)
C3G	0.039 (4)	0.052 (5)	0.019 (4)	0.008 (4)	0.004 (3)	0.004 (3)
C3H	0.030 (4)	0.039 (5)	0.035 (4)	−0.002 (3)	0.002 (3)	0.003 (4)
C4	0.027 (4)	0.042 (5)	0.047 (5)	−0.005 (3)	0.010 (3)	−0.012 (4)
C4A	0.037 (4)	0.039 (4)	0.045 (5)	0.003 (3)	0.004 (4)	−0.001 (4)
C4C	0.034 (4)	0.045 (5)	0.026 (4)	0.003 (3)	0.004 (3)	0.011 (3)
C4D	0.042 (4)	0.025 (4)	0.033 (4)	0.005 (3)	0.014 (3)	0.002 (3)
C4E	0.042 (4)	0.032 (4)	0.029 (4)	−0.007 (3)	0.004 (3)	0.008 (3)
C4F	0.022 (3)	0.043 (5)	0.036 (4)	0.003 (3)	0.006 (3)	−0.002 (4)
C4G	0.041 (4)	0.058 (6)	0.019 (4)	0.005 (4)	0.008 (3)	−0.005 (4)
C4H	0.027 (4)	0.024 (4)	0.040 (4)	−0.005 (3)	−0.002 (3)	−0.004 (3)
C5	0.044 (5)	0.076 (7)	0.028 (4)	−0.027 (5)	0.001 (4)	0.009 (4)
C5C	0.029 (4)	0.056 (5)	0.023 (4)	0.001 (4)	0.004 (3)	0.006 (4)
C5D	0.047 (5)	0.042 (5)	0.026 (4)	−0.003 (4)	0.007 (4)	−0.002 (4)
C5E	0.043 (4)	0.037 (5)	0.032 (4)	0.002 (4)	−0.002 (3)	0.008 (4)
C5F	0.026 (4)	0.049 (5)	0.038 (5)	0.008 (3)	−0.004 (3)	0.000 (4)
C5G	0.025 (4)	0.061 (6)	0.024 (4)	0.006 (4)	0.006 (3)	−0.004 (4)
C5H	0.035 (4)	0.023 (4)	0.041 (5)	−0.001 (3)	−0.006 (3)	−0.010 (3)
C05L	0.027 (4)	0.046 (5)	0.043 (5)	0.008 (4)	0.004 (3)	−0.002 (4)
C6	0.030 (4)	0.082 (7)	0.041 (5)	−0.024 (4)	0.016 (4)	−0.023 (5)
C6C	0.035 (4)	0.053 (5)	0.033 (4)	0.001 (4)	−0.005 (3)	−0.008 (4)
C6D	0.038 (4)	0.040 (5)	0.041 (5)	−0.001 (4)	0.008 (4)	−0.001 (4)
C6E	0.051 (5)	0.031 (5)	0.044 (5)	−0.007 (4)	−0.009 (4)	0.010 (4)
C6F	0.026 (4)	0.065 (6)	0.030 (4)	−0.001 (4)	0.003 (3)	0.016 (4)
C6G	0.038 (4)	0.045 (5)	0.033 (4)	0.007 (4)	0.005 (4)	0.008 (4)
C6H	0.067 (6)	0.026 (4)	0.029 (4)	−0.004 (4)	0.000 (4)	−0.001 (3)
C7	0.031 (4)	0.042 (5)	0.023 (4)	0.003 (3)	0.008 (3)	−0.006 (3)
C7A	0.038 (4)	0.033 (4)	0.038 (5)	0.000 (3)	0.009 (4)	−0.002 (4)
C7B	0.034 (4)	0.056 (6)	0.046 (5)	−0.012 (4)	0.007 (4)	0.012 (5)
C7C	0.041 (5)	0.055 (6)	0.039 (5)	0.000 (4)	0.012 (4)	0.009 (4)
C7D	0.048 (5)	0.031 (4)	0.033 (4)	0.002 (4)	−0.003 (4)	0.005 (3)
C7E	0.050 (5)	0.042 (5)	0.027 (4)	−0.002 (4)	0.004 (4)	0.010 (4)
C7G	0.036 (4)	0.062 (6)	0.022 (4)	−0.004 (4)	0.007 (3)	−0.012 (4)
C7H	0.022 (3)	0.033 (4)	0.046 (5)	−0.001 (3)	−0.002 (3)	−0.005 (4)
C8	0.032 (4)	0.034 (4)	0.032 (4)	0.008 (3)	0.000 (3)	−0.009 (3)
C8A	0.033 (4)	0.064 (6)	0.052 (6)	−0.002 (4)	0.013 (4)	−0.007 (5)
C9	0.032 (4)	0.041 (5)	0.037 (4)	−0.012 (3)	0.002 (3)	0.005 (4)
C12	0.031 (4)	0.048 (5)	0.028 (4)	−0.007 (3)	0.006 (3)	−0.005 (4)
C12A	0.040 (5)	0.046 (5)	0.058 (6)	−0.005 (4)	0.015 (4)	0.008 (5)
C17A	0.048 (5)	0.027 (4)	0.032 (4)	−0.007 (3)	−0.005 (3)	−0.007 (3)
C18A	0.073 (7)	0.042 (5)	0.043 (5)	−0.023 (5)	0.001 (5)	−0.008 (4)
C22A	0.078 (8)	0.052 (7)	0.076 (8)	0.016 (6)	−0.015 (6)	−0.029 (6)
C26A	0.067 (6)	0.056 (6)	0.027 (4)	−0.009 (5)	−0.002 (4)	−0.015 (4)
C31A	0.021 (3)	0.027 (4)	0.032 (4)	0.006 (3)	0.005 (3)	−0.004 (3)
C32A	0.042 (5)	0.046 (5)	0.043 (5)	0.002 (4)	0.016 (4)	−0.012 (4)
C36A	0.031 (4)	0.037 (4)	0.031 (4)	0.003 (3)	0.004 (3)	0.002 (3)
C40A	0.036 (4)	0.031 (4)	0.056 (5)	0.004 (3)	0.004 (4)	−0.012 (4)
C45A	0.039 (4)	0.033 (4)	0.027 (4)	0.012 (3)	−0.005 (3)	0.000 (3)
C46A	0.069 (7)	0.054 (6)	0.041 (5)	0.021 (5)	−0.014 (5)	−0.015 (5)
C50A	0.041 (5)	0.047 (5)	0.053 (6)	0.021 (4)	−0.008 (4)	0.013 (4)
C54A	0.062 (6)	0.045 (5)	0.057 (6)	0.027 (5)	0.026 (5)	0.023 (5)
C17C	0.026 (4)	0.036 (4)	0.018 (4)	0.004 (3)	−0.002 (3)	0.008 (3)
C26C	0.037 (7)	0.075 (11)	0.036 (6)	−0.004 (6)	−0.006 (5)	0.011 (7)
F29C	0.07 (2)	0.15 (3)	0.039 (18)	−0.02 (3)	−0.030 (17)	0.02 (2)
F27C	0.074 (12)	0.065 (12)	0.079 (13)	−0.004 (10)	−0.019 (10)	0.011 (11)
F28C	0.085 (13)	0.078 (13)	0.048 (11)	0.022 (11)	0.013 (10)	−0.012 (10)
F21C	0.045 (3)	0.035 (5)	0.039 (5)	−0.013 (3)	−0.012 (3)	0.014 (3)
C18C	0.032 (5)	0.041 (5)	0.030 (6)	−0.005 (4)	−0.013 (4)	0.008 (5)
F20C	0.11 (2)	0.060 (16)	0.052 (15)	−0.008 (15)	−0.012 (14)	−0.018 (13)
F19C	0.06 (3)	0.05 (3)	0.09 (3)	0.004 (18)	0.00 (2)	0.03 (2)
C22C	0.037 (5)	0.039 (8)	0.075 (8)	0.001 (5)	−0.001 (5)	−0.011 (6)
F23C	0.036 (12)	0.039 (15)	0.13 (3)	0.004 (11)	−0.012 (16)	0.000 (18)
F25C	0.031 (3)	0.043 (4)	0.090 (7)	0.005 (3)	0.017 (4)	0.013 (4)
F24C	0.046 (14)	0.13 (3)	0.13 (3)	−0.019 (15)	0.049 (16)	−0.08 (2)

### Tetrakis(perfluorobenzonitrile)silver(I) tetrakis(perfluoro-tert-butoxy)aluminate(1-) Geometric parameters (Å, º)

**Table:**

Ag01—N1E	2.272 (8)	F20A—C18A	1.349 (11)
Ag01—N1F	2.383 (8)	F21A—C18A	1.352 (12)
Ag01—N1G	2.238 (9)	F23A—C22A	1.379 (16)
Ag01—N1H	2.280 (8)	F24A—C22A	1.349 (12)
Ag02—N1A	2.240 (7)	F25A—C22A	1.298 (14)
Ag02—N1B	2.371 (9)	F27A—C26A	1.318 (11)
Ag02—N1C	2.207 (8)	F28A—C26A	1.337 (12)
Ag02—N1D	2.252 (7)	F29A—C26A	1.361 (10)
Al1A—O2A	1.724 (6)	F33A—C32A	1.303 (10)
Al1A—O16A	1.737 (5)	F34A—C32A	1.349 (10)
Al1A—O30A	1.726 (5)	F35A—C32A	1.334 (10)
Al1A—O44A	1.718 (5)	F37A—C36A	1.343 (9)
Al1B—O2B	1.722 (5)	F38A—C36A	1.354 (9)
Al1B—O16B	1.721 (5)	F39A—C36A	1.317 (9)
Al1B—O30B	1.723 (5)	F41A—C40A	1.331 (10)
Al1B—O44B	1.705 (5)	F42A—C40A	1.366 (11)
F5B—C4B	1.328 (10)	F43A—C40A	1.322 (10)
F6B—C4B	1.319 (9)	F47A—C46A	1.317 (11)
F7B—C4B	1.343 (10)	F48A—C46A	1.363 (13)
F9B—C8B	1.349 (10)	F49A—C46A	1.255 (14)
F10B—C8B	1.324 (10)	F51A—C50A	1.287 (12)
F11B—C8B	1.334 (9)	F52A—C50A	1.333 (11)
F13B—C12B	1.339 (10)	F53A—C50A	1.280 (12)
F14B—C12B	1.318 (10)	F55A—C54A	1.345 (13)
F15B—C12B	1.345 (9)	F56A—C54A	1.291 (11)
F19B—C18B	1.358 (14)	F57A—C54A	1.359 (11)
F20B—C18B	1.302 (18)	O2A—C3A	1.364 (9)
F21B—C18B	1.331 (12)	O16A—C17A	1.345 (9)
F23B—C22B	1.304 (16)	O30A—C31A	1.352 (8)
F24B—C22B	1.338 (17)	O44A—C45A	1.352 (9)
F25B—C22B	1.371 (13)	N1A—C7A	1.157 (11)
F27B—C26B	1.347 (16)	N1B—C7B	1.124 (13)
F28B—C26B	1.357 (16)	N1C—C7C	1.156 (12)
F29B—C26B	1.303 (14)	N1D—C7D	1.147 (11)
F33B—C32B	1.325 (10)	N1E—C7E	1.129 (12)
F34B—C32B	1.341 (10)	N1F—C05L	1.136 (12)
F35B—C32B	1.321 (10)	N1G—C7G	1.129 (12)
F37B—C36B	1.351 (11)	N1H—C7H	1.137 (11)
F38B—C36B	1.346 (10)	C1—C2A	1.366 (13)
F39B—C36B	1.298 (12)	C1—C3	1.377 (13)
F41B—C40B	1.355 (9)	C1C—C2C	1.380 (13)
F42B—C40B	1.341 (12)	C1C—C6C	1.382 (12)
F43B—C40B	1.311 (11)	C1D—C2D	1.361 (13)
F47B—C46B	1.361 (10)	C1D—C6D	1.388 (12)
F48B—C46B	1.293 (11)	C1E—C2E	1.380 (13)
F49B—C46B	1.342 (11)	C1E—C6E	1.380 (14)
F51B—C50B	1.335 (11)	C1F—C2F	1.401 (12)
F52B—C50B	1.344 (13)	C1F—C6F	1.359 (13)
F53B—C50B	1.327 (12)	C1G—C2G	1.366 (15)
F55B—C54B	1.361 (15)	C1G—C6G	1.391 (12)
F56B—C54B	1.306 (13)	C1H—C2H	1.381 (12)
F57B—C54B	1.321 (11)	C1H—C6H	1.420 (12)
O2B—C3B	1.350 (8)	C2—C3	1.348 (12)
O16B—C17B	1.362 (10)	C2—C8	1.407 (12)
O16B—C17C	1.32 (2)	C2A—C7	1.358 (12)
O30B—C31B	1.351 (8)	C2B—C4	1.387 (12)
O44B—C45B	1.361 (9)	C2B—C12	1.365 (12)
C3B—C4B	1.541 (11)	C2C—C3C	1.379 (12)
C3B—C8B	1.561 (11)	C2D—C3D	1.389 (13)
C3B—C12B	1.546 (10)	C2E—C3E	1.377 (12)
C17B—C18B	1.546 (14)	C2F—C3F	1.380 (12)
C17B—C22B	1.534 (14)	C2G—C3G	1.391 (14)
C17B—C26B	1.559 (14)	C2H—C3H	1.349 (12)
C31B—C32B	1.561 (11)	C3A—C4A	1.559 (11)
C31B—C36B	1.544 (11)	C3A—C8A	1.514 (12)
C31B—C40B	1.531 (12)	C3A—C12A	1.548 (12)
C45B—C46B	1.564 (12)	C3C—C4C	1.376 (12)
C45B—C50B	1.535 (13)	C3D—C4D	1.385 (12)
C45B—C54B	1.541 (13)	C3E—C4E	1.394 (12)
F00S—C2F	1.315 (9)	C3F—C4F	1.390 (12)
F1—C2	1.340 (9)	C3G—C4G	1.389 (12)
F1A—C1	1.345 (9)	C3H—C4H	1.390 (11)
F1B—C4	1.311 (11)	C4—C6	1.394 (14)
F1C—C1C	1.331 (10)	C4C—C5C	1.400 (13)
F1D—C1D	1.322 (10)	C4C—C7C	1.409 (13)
F1E—C1E	1.309 (10)	C4D—C5D	1.400 (12)
F1F—C1F	1.318 (10)	C4D—C7D	1.425 (12)
F1G—C1G	1.325 (11)	C4E—C5E	1.386 (12)
F1H—C1H	1.327 (9)	C4E—C7E	1.438 (12)
F2—C3	1.341 (10)	C4F—C5F	1.380 (12)
F2A—C2A	1.334 (10)	C4F—C05L	1.444 (13)
F2B—C2B	1.324 (10)	C4G—C5G	1.380 (13)
F2C—C2C	1.320 (9)	C4G—C7G	1.436 (13)
F2D—C2D	1.344 (10)	C4H—C5H	1.414 (12)
F2E—C2E	1.326 (10)	C4H—C7H	1.427 (12)
F2G—C2G	1.332 (10)	C5—C6	1.352 (15)
F2H—C2H	1.338 (10)	C5—C9	1.388 (13)
F3A—C7	1.336 (10)	C5C—C6C	1.352 (13)
F3B—C12	1.326 (9)	C5D—C6D	1.380 (12)
F3C—C3C	1.334 (11)	C5E—C6E	1.382 (12)
F3D—C3D	1.338 (10)	C5F—C6F	1.392 (13)
F3E—C3E	1.315 (9)	C5G—C6G	1.381 (13)
F3F—C3F	1.318 (9)	C5H—C6H	1.351 (13)
F3G—C3G	1.320 (10)	C7—C8	1.397 (11)
F3H—C3H	1.338 (10)	C7A—C8	1.406 (12)
F5A—C4A	1.322 (10)	C7B—C9	1.448 (14)
F5C—C5C	1.342 (9)	C9—C12	1.398 (12)
F5D—C5D	1.326 (10)	C17A—C18A	1.531 (14)
F5E—C5E	1.326 (10)	C17A—C22A	1.524 (14)
F5F—C5F	1.307 (10)	C17A—C26A	1.553 (13)
F5G—C5G	1.332 (9)	C31A—C32A	1.553 (12)
F5H—C5H	1.306 (9)	C31A—C36A	1.555 (10)
F6A—C4A	1.317 (10)	C31A—C40A	1.529 (11)
F6C—C6C	1.345 (11)	C45A—C46A	1.557 (12)
F6D—C6D	1.323 (10)	C45A—C50A	1.552 (11)
F6E—C6E	1.308 (11)	C45A—C54A	1.547 (13)
F6F—C6F	1.340 (10)	C17C—C26C	1.54 (3)
F6G—C6G	1.327 (10)	C17C—C18C	1.54 (2)
F6H—C6H	1.319 (10)	C17C—C22C	1.54 (2)
F7A—C4A	1.357 (10)	C26C—F29C	1.31 (3)
F9A—C8A	1.369 (12)	C26C—F27C	1.35 (3)
F10A—C8A	1.370 (11)	C26C—F28C	1.37 (3)
F11A—C8A	1.325 (11)	F21C—C18C	1.34 (2)
F13A—C12A	1.360 (11)	C18C—F20C	1.30 (3)
F14A—C12A	1.350 (10)	C18C—F19C	1.35 (3)
F15A—C12A	1.296 (12)	C22C—F23C	1.31 (3)
F18—C5	1.336 (11)	C22C—F25C	1.37 (2)
F19A—C18A	1.345 (13)	C22C—F24C	1.36 (3)
F20—C6	1.333 (11)		
			
N1E—Ag01—N1F	94.5 (3)	C2F—C3F—C4F	120.4 (7)
N1E—Ag01—N1H	110.0 (3)	F3G—C3G—C2G	119.9 (8)
N1G—Ag01—N1E	120.3 (3)	F3G—C3G—C4G	120.2 (8)
N1G—Ag01—N1F	113.0 (3)	C4G—C3G—C2G	119.8 (8)
N1G—Ag01—N1H	113.2 (3)	F3H—C3H—C2H	120.9 (7)
N1H—Ag01—N1F	103.1 (3)	F3H—C3H—C4H	117.5 (7)
N1A—Ag02—N1B	98.1 (3)	C2H—C3H—C4H	121.5 (8)
N1A—Ag02—N1D	110.3 (3)	F1B—C4—C2B	119.8 (9)
N1C—Ag02—N1A	119.7 (3)	F1B—C4—C6	120.9 (8)
N1C—Ag02—N1B	110.0 (3)	C2B—C4—C6	119.3 (8)
N1C—Ag02—N1D	113.9 (3)	F5A—C4A—F7A	107.7 (7)
N1D—Ag02—N1B	102.3 (3)	F5A—C4A—C3A	110.1 (6)
O2A—Al1A—O16A	107.0 (3)	F6A—C4A—F5A	109.0 (7)
O2A—Al1A—O30A	112.8 (3)	F6A—C4A—F7A	107.6 (7)
O30A—Al1A—O16A	108.3 (3)	F6A—C4A—C3A	112.7 (7)
O44A—Al1A—O2A	106.8 (3)	F7A—C4A—C3A	109.5 (7)
O44A—Al1A—O16A	114.9 (3)	C3C—C4C—C5C	117.8 (8)
O44A—Al1A—O30A	107.2 (3)	C3C—C4C—C7C	121.9 (9)
O2B—Al1B—O30B	107.3 (3)	C5C—C4C—C7C	120.2 (8)
O16B—Al1B—O2B	107.6 (3)	C3D—C4D—C5D	118.4 (7)
O16B—Al1B—O30B	110.7 (3)	C3D—C4D—C7D	120.6 (8)
O44B—Al1B—O2B	114.1 (3)	C5D—C4D—C7D	120.9 (8)
O44B—Al1B—O16B	109.0 (3)	C3E—C4E—C7E	121.0 (8)
O44B—Al1B—O30B	108.2 (3)	C5E—C4E—C3E	119.1 (8)
C3B—O2B—Al1B	148.9 (5)	C5E—C4E—C7E	119.8 (8)
C17B—O16B—Al1B	145.3 (5)	C3F—C4F—C05L	118.4 (8)
C17C—O16B—Al1B	156.4 (11)	C5F—C4F—C3F	120.2 (8)
C31B—O30B—Al1B	147.7 (5)	C5F—C4F—C05L	121.1 (8)
C45B—O44B—Al1B	149.9 (5)	C3G—C4G—C7G	120.4 (8)
O2B—C3B—C4B	111.5 (6)	C5G—C4G—C3G	119.3 (8)
O2B—C3B—C8B	107.8 (6)	C5G—C4G—C7G	120.1 (8)
O2B—C3B—C12B	110.6 (6)	C3H—C4H—C5H	117.6 (7)
C4B—C3B—C8B	108.0 (6)	C3H—C4H—C7H	121.3 (8)
C4B—C3B—C12B	109.5 (6)	C5H—C4H—C7H	120.9 (7)
C12B—C3B—C8B	109.4 (6)	F18—C5—C6	119.7 (9)
F5B—C4B—F7B	107.5 (6)	F18—C5—C9	119.0 (9)
F5B—C4B—C3B	113.3 (7)	C6—C5—C9	121.3 (9)
F6B—C4B—F5B	107.5 (7)	F5C—C5C—C4C	119.1 (8)
F6B—C4B—F7B	106.2 (7)	F5C—C5C—C6C	120.1 (8)
F6B—C4B—C3B	111.1 (6)	C6C—C5C—C4C	120.8 (8)
F7B—C4B—C3B	110.9 (7)	F5D—C5D—C4D	119.1 (7)
F9B—C8B—C3B	110.0 (7)	F5D—C5D—C6D	120.3 (8)
F10B—C8B—F9B	107.0 (7)	C6D—C5D—C4D	120.5 (8)
F10B—C8B—F11B	107.7 (7)	F5E—C5E—C4E	119.4 (8)
F10B—C8B—C3B	111.6 (7)	F5E—C5E—C6E	119.6 (8)
F11B—C8B—F9B	107.2 (7)	C6E—C5E—C4E	121.0 (8)
F11B—C8B—C3B	113.1 (7)	F5F—C5F—C4F	120.7 (8)
F13B—C12B—F15B	107.4 (6)	F5F—C5F—C6F	120.0 (8)
F13B—C12B—C3B	110.3 (6)	C4F—C5F—C6F	119.2 (8)
F14B—C12B—F13B	107.8 (7)	F5G—C5G—C4G	120.1 (8)
F14B—C12B—F15B	107.9 (7)	F5G—C5G—C6G	118.9 (8)
F14B—C12B—C3B	111.0 (6)	C4G—C5G—C6G	121.0 (7)
F15B—C12B—C3B	112.3 (6)	F5H—C5H—C4H	118.5 (8)
O16B—C17B—C18B	114.1 (8)	F5H—C5H—C6H	120.2 (8)
O16B—C17B—C22B	109.7 (8)	C6H—C5H—C4H	121.3 (8)
O16B—C17B—C26B	107.3 (8)	N1F—C05L—C4F	176.0 (10)
C18B—C17B—C26B	106.8 (9)	F20—C6—C4	118.5 (10)
C22B—C17B—C18B	110.9 (9)	F20—C6—C5	121.2 (10)
C22B—C17B—C26B	107.7 (9)	C5—C6—C4	120.3 (8)
F19B—C18B—C17B	109.8 (9)	F6C—C6C—C1C	120.2 (8)
F20B—C18B—F19B	106.4 (10)	F6C—C6C—C5C	119.5 (8)
F20B—C18B—F21B	109.4 (12)	C5C—C6C—C1C	120.2 (9)
F20B—C18B—C17B	111.1 (12)	F6D—C6D—C1D	119.4 (8)
F21B—C18B—F19B	104.6 (9)	F6D—C6D—C5D	120.6 (8)
F21B—C18B—C17B	115.1 (8)	C5D—C6D—C1D	120.0 (8)
F23B—C22B—F24B	107.6 (12)	F6E—C6E—C1E	120.1 (8)
F23B—C22B—F25B	108.1 (10)	F6E—C6E—C5E	120.9 (9)
F23B—C22B—C17B	114.4 (11)	C1E—C6E—C5E	118.9 (9)
F24B—C22B—F25B	105.0 (11)	F6F—C6F—C1F	119.6 (8)
F24B—C22B—C17B	109.5 (10)	F6F—C6F—C5F	119.6 (8)
F25B—C22B—C17B	111.6 (9)	C1F—C6F—C5F	120.7 (8)
F27B—C26B—F28B	105.8 (11)	F6G—C6G—C1G	119.5 (8)
F27B—C26B—C17B	110.2 (10)	F6G—C6G—C5G	121.4 (8)
F28B—C26B—C17B	110.6 (10)	C5G—C6G—C1G	119.1 (8)
F29B—C26B—F27B	107.0 (11)	F6H—C6H—C1H	117.5 (8)
F29B—C26B—F28B	107.5 (12)	F6H—C6H—C5H	122.9 (9)
F29B—C26B—C17B	115.2 (11)	C5H—C6H—C1H	119.6 (8)
O30B—C31B—C32B	107.4 (6)	F3A—C7—C2A	119.9 (8)
O30B—C31B—C36B	108.8 (6)	F3A—C7—C8	118.2 (7)
O30B—C31B—C40B	112.1 (6)	C2A—C7—C8	121.8 (8)
C36B—C31B—C32B	107.9 (6)	N1A—C7A—C8	178.9 (10)
C40B—C31B—C32B	109.8 (7)	N1B—C7B—C9	176.3 (10)
C40B—C31B—C36B	110.9 (7)	N1C—C7C—C4C	178.4 (11)
F33B—C32B—F34B	108.1 (7)	N1D—C7D—C4D	179.2 (10)
F33B—C32B—C31B	110.4 (7)	N1E—C7E—C4E	178.0 (10)
F34B—C32B—C31B	111.9 (7)	N1G—C7G—C4G	179.7 (10)
F35B—C32B—F33B	108.0 (7)	N1H—C7H—C4H	176.4 (9)
F35B—C32B—F34B	106.8 (7)	C7—C8—C2	116.4 (7)
F35B—C32B—C31B	111.3 (7)	C7—C8—C7A	121.8 (8)
F37B—C36B—C31B	108.9 (7)	C7A—C8—C2	121.8 (7)
F38B—C36B—F37B	106.5 (7)	F9A—C8A—F10A	106.6 (8)
F38B—C36B—C31B	112.3 (7)	F9A—C8A—C3A	109.6 (8)
F39B—C36B—F37B	106.9 (8)	F10A—C8A—C3A	110.7 (7)
F39B—C36B—F38B	109.0 (7)	F11A—C8A—F9A	106.4 (8)
F39B—C36B—C31B	113.0 (7)	F11A—C8A—F10A	106.9 (8)
F41B—C40B—C31B	112.5 (7)	F11A—C8A—C3A	116.1 (8)
F42B—C40B—F41B	105.6 (8)	C5—C9—C7B	122.8 (8)
F42B—C40B—C31B	111.3 (7)	C5—C9—C12	118.1 (8)
F43B—C40B—F41B	107.8 (8)	C12—C9—C7B	119.0 (8)
F43B—C40B—F42B	107.9 (8)	F3B—C12—C2B	119.5 (8)
F43B—C40B—C31B	111.4 (7)	F3B—C12—C9	119.5 (8)
O44B—C45B—C46B	109.5 (6)	C2B—C12—C9	121.0 (8)
O44B—C45B—C50B	109.2 (7)	F13A—C12A—C3A	108.6 (7)
O44B—C45B—C54B	111.4 (7)	F14A—C12A—F13A	105.3 (8)
C50B—C45B—C46B	107.5 (7)	F14A—C12A—C3A	112.1 (7)
C50B—C45B—C54B	111.2 (8)	F15A—C12A—F13A	110.3 (8)
C54B—C45B—C46B	107.9 (8)	F15A—C12A—F14A	108.2 (8)
F47B—C46B—C45B	111.3 (7)	F15A—C12A—C3A	112.1 (8)
F48B—C46B—F47B	107.7 (7)	O16A—C17A—C18A	107.9 (7)
F48B—C46B—F49B	107.0 (8)	O16A—C17A—C22A	110.6 (7)
F48B—C46B—C45B	112.4 (7)	O16A—C17A—C26A	111.0 (7)
F49B—C46B—F47B	107.5 (7)	C18A—C17A—C26A	108.0 (8)
F49B—C46B—C45B	110.8 (7)	C22A—C17A—C18A	110.3 (8)
F51B—C50B—F52B	106.7 (9)	C22A—C17A—C26A	109.1 (8)
F51B—C50B—C45B	113.3 (9)	F19A—C18A—F20A	108.6 (8)
F52B—C50B—C45B	111.5 (8)	F19A—C18A—F21A	105.5 (9)
F53B—C50B—F51B	106.9 (8)	F19A—C18A—C17A	111.7 (8)
F53B—C50B—F52B	106.7 (9)	F20A—C18A—F21A	105.2 (8)
F53B—C50B—C45B	111.4 (8)	F20A—C18A—C17A	114.3 (9)
F55B—C54B—C45B	108.4 (10)	F21A—C18A—C17A	110.9 (8)
F56B—C54B—F55B	107.3 (9)	F23A—C22A—C17A	109.0 (10)
F56B—C54B—F57B	109.9 (10)	F24A—C22A—F23A	107.3 (10)
F56B—C54B—C45B	112.2 (8)	F24A—C22A—C17A	114.1 (9)
F57B—C54B—F55B	106.1 (9)	F25A—C22A—F23A	105.7 (10)
F57B—C54B—C45B	112.6 (8)	F25A—C22A—F24A	108.0 (10)
C3A—O2A—Al1A	148.3 (5)	F25A—C22A—C17A	112.3 (9)
C17A—O16A—Al1A	146.7 (5)	F27A—C26A—F28A	109.7 (9)
C31A—O30A—Al1A	148.0 (5)	F27A—C26A—F29A	106.3 (8)
C45A—O44A—Al1A	149.1 (5)	F27A—C26A—C17A	111.2 (7)
C7A—N1A—Ag02	171.7 (7)	F28A—C26A—F29A	107.2 (8)
C7B—N1B—Ag02	144.8 (8)	F28A—C26A—C17A	110.9 (8)
C7C—N1C—Ag02	173.1 (7)	F29A—C26A—C17A	111.3 (8)
C7D—N1D—Ag02	177.0 (7)	O30A—C31A—C32A	110.4 (6)
C7E—N1E—Ag01	174.8 (8)	O30A—C31A—C36A	111.3 (6)
C05L—N1F—Ag01	148.3 (7)	O30A—C31A—C40A	108.1 (6)
C7G—N1G—Ag01	165.4 (7)	C32A—C31A—C36A	107.9 (6)
C7H—N1H—Ag01	173.4 (7)	C40A—C31A—C32A	109.4 (7)
F1A—C1—C2A	119.5 (8)	C40A—C31A—C36A	109.6 (6)
F1A—C1—C3	119.4 (8)	F33A—C32A—F34A	106.2 (8)
C2A—C1—C3	121.1 (8)	F33A—C32A—F35A	109.2 (7)
F1C—C1C—C2C	120.3 (8)	F33A—C32A—C31A	112.3 (7)
F1C—C1C—C6C	118.9 (8)	F34A—C32A—C31A	109.0 (7)
C2C—C1C—C6C	120.8 (8)	F35A—C32A—F34A	107.3 (7)
F1D—C1D—C2D	120.6 (8)	F35A—C32A—C31A	112.6 (7)
F1D—C1D—C6D	119.4 (8)	F37A—C36A—F38A	106.9 (6)
C2D—C1D—C6D	120.0 (8)	F37A—C36A—C31A	112.0 (6)
F1E—C1E—C2E	119.7 (9)	F38A—C36A—C31A	111.7 (6)
F1E—C1E—C6E	119.2 (9)	F39A—C36A—F37A	107.8 (6)
C6E—C1E—C2E	121.1 (8)	F39A—C36A—F38A	107.9 (6)
F1F—C1F—C2F	119.1 (8)	F39A—C36A—C31A	110.4 (6)
F1F—C1F—C6F	120.4 (8)	F41A—C40A—F42A	105.0 (7)
C6F—C1F—C2F	120.5 (8)	F41A—C40A—C31A	112.2 (7)
F1G—C1G—C2G	119.8 (8)	F42A—C40A—C31A	109.2 (7)
F1G—C1G—C6G	119.8 (10)	F43A—C40A—F41A	107.3 (7)
C2G—C1G—C6G	120.4 (9)	F43A—C40A—F42A	107.3 (7)
F1H—C1H—C2H	121.4 (7)	F43A—C40A—C31A	115.3 (7)
F1H—C1H—C6H	119.7 (8)	O44A—C45A—C46A	111.4 (7)
C2H—C1H—C6H	118.9 (7)	O44A—C45A—C50A	107.5 (6)
F1—C2—C3	119.9 (8)	O44A—C45A—C54A	110.5 (7)
F1—C2—C8	118.2 (8)	C50A—C45A—C46A	111.0 (7)
C3—C2—C8	121.9 (8)	C54A—C45A—C46A	107.5 (8)
F2A—C2A—C1	120.3 (8)	C54A—C45A—C50A	108.9 (7)
F2A—C2A—C7	120.4 (9)	F47A—C46A—F48A	105.0 (8)
C7—C2A—C1	119.3 (8)	F47A—C46A—C45A	112.9 (8)
F2B—C2B—C4	119.0 (8)	F48A—C46A—C45A	110.2 (9)
F2B—C2B—C12	121.1 (7)	F49A—C46A—F47A	109.6 (10)
C12—C2B—C4	119.9 (8)	F49A—C46A—F48A	107.8 (10)
F2C—C2C—C1C	120.5 (8)	F49A—C46A—C45A	111.1 (9)
F2C—C2C—C3C	121.4 (8)	F51A—C50A—F52A	103.2 (9)
C3C—C2C—C1C	118.0 (8)	F51A—C50A—C45A	109.2 (8)
F2D—C2D—C1D	119.9 (8)	F52A—C50A—C45A	111.9 (8)
F2D—C2D—C3D	119.5 (8)	F53A—C50A—F51A	113.5 (10)
C1D—C2D—C3D	120.5 (8)	F53A—C50A—F52A	107.2 (8)
F2E—C2E—C1E	119.9 (8)	F53A—C50A—C45A	111.5 (7)
F2E—C2E—C3E	120.3 (8)	F55A—C54A—F57A	100.2 (9)
C3E—C2E—C1E	119.8 (8)	F55A—C54A—C45A	112.1 (7)
F00S—C2F—C1F	119.9 (8)	F56A—C54A—F55A	104.3 (8)
F00S—C2F—C3F	121.1 (8)	F56A—C54A—F57A	109.8 (8)
C3F—C2F—C1F	118.9 (8)	F56A—C54A—C45A	116.3 (10)
F2G—C2G—C1G	119.4 (9)	F57A—C54A—C45A	112.6 (7)
F2G—C2G—C3G	120.1 (9)	O16B—C17C—C26C	110.0 (19)
C1G—C2G—C3G	120.3 (8)	O16B—C17C—C18C	108.4 (17)
F2H—C2H—C1H	117.4 (7)	O16B—C17C—C22C	107.9 (18)
F2H—C2H—C3H	121.7 (8)	C26C—C17C—C18C	109 (2)
C3H—C2H—C1H	121.0 (8)	C26C—C17C—C22C	112.0 (19)
F2—C3—C1	119.5 (8)	C18C—C17C—C22C	110 (2)
F2—C3—C2	121.2 (8)	F29C—C26C—C17C	114 (3)
C2—C3—C1	119.3 (8)	F29C—C26C—F27C	107 (3)
O2A—C3A—C4A	112.0 (6)	F29C—C26C—F28C	106 (3)
O2A—C3A—C8A	110.9 (7)	F27C—C26C—C17C	111 (2)
O2A—C3A—C12A	108.6 (6)	F27C—C26C—F28C	108 (3)
C8A—C3A—C4A	108.1 (6)	F28C—C26C—C17C	112 (2)
C8A—C3A—C12A	110.3 (7)	F21C—C18C—C17C	106 (2)
C12A—C3A—C4A	106.8 (7)	F21C—C18C—F19C	111 (3)
F3C—C3C—C2C	117.9 (8)	F20C—C18C—C17C	113 (2)
F3C—C3C—C4C	119.8 (8)	F20C—C18C—F21C	108 (3)
C4C—C3C—C2C	122.3 (9)	F20C—C18C—F19C	107 (3)
F3D—C3D—C2D	119.6 (8)	F19C—C18C—C17C	112 (3)
F3D—C3D—C4D	119.9 (8)	F23C—C22C—C17C	112 (3)
C4D—C3D—C2D	120.5 (8)	F23C—C22C—F25C	107 (3)
F3E—C3E—C2E	120.9 (8)	F23C—C22C—F24C	107 (3)
F3E—C3E—C4E	118.9 (7)	F25C—C22C—C17C	107 (2)
C2E—C3E—C4E	120.1 (8)	F24C—C22C—C17C	107 (2)
F3F—C3F—C2F	119.3 (8)	F24C—C22C—F25C	117 (3)
F3F—C3F—C4F	120.4 (8)		
			
Al1A—O2A—C3A—C4A	5.1 (12)	O2A—C3A—C4A—F6A	−165.9 (6)
Al1A—O2A—C3A—C8A	125.9 (9)	O2A—C3A—C4A—F7A	74.4 (8)
Al1A—O2A—C3A—C12A	−112.7 (9)	O2A—C3A—C8A—F9A	−43.6 (9)
Al1A—O16A—C17A—C18A	−168.2 (7)	O2A—C3A—C8A—F10A	73.7 (9)
Al1A—O16A—C17A—C22A	71.1 (12)	O2A—C3A—C8A—F11A	−164.2 (8)
Al1A—O16A—C17A—C26A	−50.1 (13)	O2A—C3A—C12A—F13A	−47.4 (9)
Al1A—O30A—C31A—C32A	86.1 (10)	O2A—C3A—C12A—F14A	−163.4 (7)
Al1A—O30A—C31A—C36A	−33.7 (12)	O2A—C3A—C12A—F15A	74.7 (9)
Al1A—O30A—C31A—C40A	−154.2 (8)	O16A—Al1A—O2A—C3A	−171.5 (9)
Al1A—O44A—C45A—C46A	−74.4 (13)	O16A—Al1A—O30A—C31A	−25.8 (10)
Al1A—O44A—C45A—C50A	163.8 (8)	O16A—Al1A—O44A—C45A	−78.8 (11)
Al1A—O44A—C45A—C54A	45.1 (13)	O16A—C17A—C18A—F19A	76.5 (9)
Al1B—O2B—C3B—C4B	−68.1 (11)	O16A—C17A—C18A—F20A	−159.6 (8)
Al1B—O2B—C3B—C8B	173.5 (7)	O16A—C17A—C18A—F21A	−40.9 (10)
Al1B—O2B—C3B—C12B	53.9 (12)	O16A—C17A—C22A—F23A	73.8 (10)
Al1B—O16B—C17B—C18B	−27.7 (15)	O16A—C17A—C22A—F24A	−166.3 (10)
Al1B—O16B—C17B—C22B	97.4 (12)	O16A—C17A—C22A—F25A	−43.1 (13)
Al1B—O16B—C17B—C26B	−145.9 (9)	O16A—C17A—C26A—F27A	−40.6 (10)
Al1B—O16B—C17C—C26C	−155 (2)	O16A—C17A—C26A—F28A	81.8 (9)
Al1B—O16B—C17C—C18C	−36 (4)	O16A—C17A—C26A—F29A	−158.9 (8)
Al1B—O16B—C17C—C22C	83 (3)	O30A—Al1A—O2A—C3A	69.7 (9)
Al1B—O30B—C31B—C32B	129.5 (8)	O30A—Al1A—O16A—C17A	154.3 (9)
Al1B—O30B—C31B—C36B	−114.0 (9)	O30A—Al1A—O44A—C45A	41.6 (11)
Al1B—O30B—C31B—C40B	8.9 (13)	O30A—C31A—C32A—F33A	−46.8 (9)
Al1B—O44B—C45B—C46B	−61.8 (13)	O30A—C31A—C32A—F34A	70.6 (8)
Al1B—O44B—C45B—C50B	−179.2 (8)	O30A—C31A—C32A—F35A	−170.5 (7)
Al1B—O44B—C45B—C54B	57.5 (14)	O30A—C31A—C36A—F37A	82.6 (7)
O2B—Al1B—O16B—C17B	−25.1 (11)	O30A—C31A—C36A—F38A	−157.5 (6)
O2B—Al1B—O16B—C17C	−37 (3)	O30A—C31A—C36A—F39A	−37.4 (9)
O2B—Al1B—O30B—C31B	−168.7 (9)	O30A—C31A—C40A—F41A	−40.3 (9)
O2B—Al1B—O44B—C45B	−76.1 (11)	O30A—C31A—C40A—F42A	75.7 (8)
O2B—C3B—C4B—F5B	−161.6 (6)	O30A—C31A—C40A—F43A	−163.5 (7)
O2B—C3B—C4B—F6B	−40.4 (9)	O44A—Al1A—O2A—C3A	−47.9 (10)
O2B—C3B—C4B—F7B	77.5 (8)	O44A—Al1A—O16A—C17A	−85.9 (10)
O2B—C3B—C8B—F9B	−42.4 (8)	O44A—Al1A—O30A—C31A	−150.3 (9)
O2B—C3B—C8B—F10B	76.1 (8)	O44A—C45A—C46A—F47A	−167.5 (8)
O2B—C3B—C8B—F11B	−162.2 (7)	O44A—C45A—C46A—F48A	75.4 (10)
O2B—C3B—C12B—F13B	73.9 (8)	O44A—C45A—C46A—F49A	−43.9 (11)
O2B—C3B—C12B—F14B	−45.5 (9)	O44A—C45A—C50A—F51A	76.3 (10)
O2B—C3B—C12B—F15B	−166.4 (6)	O44A—C45A—C50A—F52A	−170.0 (8)
O16B—Al1B—O2B—C3B	161.5 (9)	O44A—C45A—C50A—F53A	−50.0 (10)
O16B—Al1B—O30B—C31B	74.2 (10)	O44A—C45A—C54A—F55A	69.0 (9)
O16B—Al1B—O44B—C45B	44.1 (11)	O44A—C45A—C54A—F56A	−171.1 (8)
O16B—C17B—C18B—F19B	81.0 (10)	O44A—C45A—C54A—F57A	−43.1 (11)
O16B—C17B—C18B—F20B	−36.4 (14)	C1—C2A—C7—F3A	−178.4 (8)
O16B—C17B—C18B—F21B	−161.4 (10)	C1—C2A—C7—C8	−1.9 (12)
O16B—C17B—C22B—F23B	71.7 (13)	C1C—C2C—C3C—F3C	179.7 (7)
O16B—C17B—C22B—F24B	−49.2 (12)	C1C—C2C—C3C—C4C	−0.9 (12)
O16B—C17B—C22B—F25B	−165.1 (10)	C1D—C2D—C3D—F3D	177.4 (8)
O16B—C17B—C26B—F27B	−39.9 (13)	C1D—C2D—C3D—C4D	−2.0 (14)
O16B—C17B—C26B—F28B	76.7 (12)	C1E—C2E—C3E—F3E	−178.9 (7)
O16B—C17B—C26B—F29B	−161.1 (11)	C1E—C2E—C3E—C4E	1.4 (12)
O16B—C17C—C26C—F29C	160 (3)	C1F—C2F—C3F—F3F	177.8 (7)
O16B—C17C—C26C—F27C	−79 (3)	C1F—C2F—C3F—C4F	−3.3 (11)
O16B—C17C—C26C—F28C	41 (3)	C1G—C2G—C3G—F3G	179.6 (8)
O16B—C17C—C18C—F21C	159 (2)	C1G—C2G—C3G—C4G	−4.4 (12)
O16B—C17C—C18C—F20C	−83 (3)	C1H—C2H—C3H—F3H	179.7 (8)
O16B—C17C—C18C—F19C	38 (3)	C1H—C2H—C3H—C4H	3.3 (13)
O16B—C17C—C22C—F23C	45 (3)	C2A—C1—C3—F2	−178.8 (8)
O16B—C17C—C22C—F25C	163 (2)	C2A—C1—C3—C2	−0.3 (12)
O16B—C17C—C22C—F24C	−72 (3)	C2A—C7—C8—C2	2.6 (11)
O30B—Al1B—O2B—C3B	42.3 (10)	C2A—C7—C8—C7A	−176.0 (8)
O30B—Al1B—O16B—C17B	91.9 (10)	C2B—C4—C6—F20	178.9 (7)
O30B—Al1B—O16B—C17C	80 (3)	C2B—C4—C6—C5	−1.6 (12)
O30B—Al1B—O44B—C45B	164.6 (10)	C2C—C1C—C6C—F6C	177.8 (8)
O30B—C31B—C32B—F33B	−47.4 (9)	C2C—C1C—C6C—C5C	0.8 (13)
O30B—C31B—C32B—F34B	−167.9 (6)	C2C—C3C—C4C—C5C	1.3 (12)
O30B—C31B—C32B—F35B	72.6 (8)	C2C—C3C—C4C—C7C	−178.4 (8)
O30B—C31B—C36B—F37B	79.7 (9)	C2D—C1D—C6D—F6D	−178.4 (8)
O30B—C31B—C36B—F38B	−162.7 (7)	C2D—C1D—C6D—C5D	−1.4 (13)
O30B—C31B—C36B—F39B	−38.9 (9)	C2D—C3D—C4D—C5D	2.4 (13)
O30B—C31B—C40B—F41B	−167.4 (7)	C2D—C3D—C4D—C7D	179.2 (8)
O30B—C31B—C40B—F42B	74.4 (9)	C2E—C1E—C6E—F6E	175.9 (8)
O30B—C31B—C40B—F43B	−46.2 (10)	C2E—C1E—C6E—C5E	0.0 (13)
O44B—Al1B—O2B—C3B	−77.5 (10)	C2E—C3E—C4E—C5E	−2.5 (12)
O44B—Al1B—O16B—C17B	−149.3 (10)	C2E—C3E—C4E—C7E	179.1 (7)
O44B—Al1B—O16B—C17C	−161 (3)	C2F—C1F—C6F—F6F	−179.8 (7)
O44B—Al1B—O30B—C31B	−45.2 (10)	C2F—C1F—C6F—C5F	−0.7 (12)
O44B—C45B—C46B—F47B	−163.0 (7)	C2F—C3F—C4F—C5F	3.5 (11)
O44B—C45B—C46B—F48B	−42.1 (10)	C2F—C3F—C4F—C05L	177.8 (7)
O44B—C45B—C46B—F49B	77.5 (9)	C2G—C1G—C6G—F6G	178.2 (8)
O44B—C45B—C50B—F51B	−164.5 (8)	C2G—C1G—C6G—C5G	−0.1 (13)
O44B—C45B—C50B—F52B	75.1 (9)	C2G—C3G—C4G—C5G	3.0 (11)
O44B—C45B—C50B—F53B	−43.9 (11)	C2G—C3G—C4G—C7G	178.3 (7)
O44B—C45B—C54B—F55B	78.2 (9)	C2H—C1H—C6H—F6H	−178.9 (8)
O44B—C45B—C54B—F56B	−40.1 (12)	C2H—C1H—C6H—C5H	0.3 (12)
O44B—C45B—C54B—F57B	−164.7 (9)	C2H—C3H—C4H—C5H	−1.6 (12)
C4B—C3B—C8B—F9B	−163.1 (6)	C2H—C3H—C4H—C7H	−178.3 (8)
C4B—C3B—C8B—F10B	−44.5 (8)	C3—C1—C2A—F2A	178.8 (8)
C4B—C3B—C8B—F11B	77.2 (8)	C3—C1—C2A—C7	0.7 (13)
C4B—C3B—C12B—F13B	−162.8 (6)	C3—C2—C8—C7	−2.2 (12)
C4B—C3B—C12B—F14B	77.7 (8)	C3—C2—C8—C7A	176.5 (8)
C4B—C3B—C12B—F15B	−43.1 (9)	C3C—C4C—C5C—F5C	178.8 (7)
C8B—C3B—C4B—F5B	−43.3 (8)	C3C—C4C—C5C—C6C	−0.6 (12)
C8B—C3B—C4B—F6B	77.8 (8)	C3D—C4D—C5D—F5D	178.7 (8)
C8B—C3B—C4B—F7B	−164.3 (6)	C3D—C4D—C5D—C6D	−2.3 (13)
C8B—C3B—C12B—F13B	−44.7 (8)	C3E—C4E—C5E—F5E	−178.2 (7)
C8B—C3B—C12B—F14B	−164.1 (7)	C3E—C4E—C5E—C6E	2.5 (12)
C8B—C3B—C12B—F15B	75.0 (8)	C3F—C4F—C5F—F5F	180.0 (7)
C12B—C3B—C4B—F5B	75.7 (8)	C3F—C4F—C5F—C6F	−2.3 (11)
C12B—C3B—C4B—F6B	−163.1 (6)	C3G—C4G—C5G—F5G	179.0 (7)
C12B—C3B—C4B—F7B	−45.2 (8)	C3G—C4G—C5G—C6G	−0.2 (12)
C12B—C3B—C8B—F9B	77.8 (8)	C3H—C4H—C5H—F5H	179.3 (7)
C12B—C3B—C8B—F10B	−163.6 (7)	C3H—C4H—C5H—C6H	−0.8 (12)
C12B—C3B—C8B—F11B	−41.9 (9)	C4—C2B—C12—F3B	−178.4 (7)
C18B—C17B—C22B—F23B	−161.3 (11)	C4—C2B—C12—C9	−0.5 (12)
C18B—C17B—C22B—F24B	77.7 (11)	C4A—C3A—C8A—F9A	79.5 (8)
C18B—C17B—C22B—F25B	−38.2 (14)	C4A—C3A—C8A—F10A	−163.1 (7)
C18B—C17B—C26B—F27B	−162.7 (10)	C4A—C3A—C8A—F11A	−41.0 (11)
C18B—C17B—C26B—F28B	−46.1 (12)	C4A—C3A—C12A—F13A	−168.4 (7)
C18B—C17B—C26B—F29B	76.1 (14)	C4A—C3A—C12A—F14A	75.6 (9)
C22B—C17B—C18B—F19B	−43.5 (11)	C4A—C3A—C12A—F15A	−46.3 (9)
C22B—C17B—C18B—F20B	−160.9 (11)	C4C—C5C—C6C—F6C	−177.4 (8)
C22B—C17B—C18B—F21B	74.1 (14)	C4C—C5C—C6C—C1C	−0.4 (13)
C22B—C17B—C26B—F27B	78.1 (12)	C4D—C5D—C6D—F6D	178.9 (8)
C22B—C17B—C26B—F28B	−165.2 (10)	C4D—C5D—C6D—C1D	1.8 (14)
C22B—C17B—C26B—F29B	−43.1 (16)	C4E—C5E—C6E—F6E	−177.0 (8)
C26B—C17B—C18B—F19B	−160.6 (8)	C4E—C5E—C6E—C1E	−1.2 (13)
C26B—C17B—C18B—F20B	82.0 (12)	C4F—C5F—C6F—F6F	180.0 (7)
C26B—C17B—C18B—F21B	−43.0 (14)	C4F—C5F—C6F—C1F	0.9 (12)
C26B—C17B—C22B—F23B	−44.8 (14)	C4G—C5G—C6G—F6G	−179.5 (8)
C26B—C17B—C22B—F24B	−165.7 (10)	C4G—C5G—C6G—C1G	−1.2 (12)
C26B—C17B—C22B—F25B	78.4 (13)	C4H—C5H—C6H—F6H	−179.5 (7)
C32B—C31B—C36B—F37B	−164.2 (7)	C4H—C5H—C6H—C1H	1.4 (12)
C32B—C31B—C36B—F38B	−46.5 (10)	C5—C9—C12—F3B	178.7 (7)
C32B—C31B—C36B—F39B	77.3 (8)	C5—C9—C12—C2B	0.9 (12)
C32B—C31B—C40B—F41B	73.4 (10)	C05L—C4F—C5F—F5F	5.9 (12)
C32B—C31B—C40B—F42B	−44.8 (9)	C05L—C4F—C5F—C6F	−176.4 (7)
C32B—C31B—C40B—F43B	−165.4 (7)	C6—C5—C9—C7B	−179.0 (9)
C36B—C31B—C32B—F33B	−164.5 (7)	C6—C5—C9—C12	−1.6 (13)
C36B—C31B—C32B—F34B	75.0 (9)	C6C—C1C—C2C—F2C	177.4 (7)
C36B—C31B—C32B—F35B	−44.4 (9)	C6C—C1C—C2C—C3C	−0.2 (12)
C36B—C31B—C40B—F41B	−45.6 (10)	C6D—C1D—C2D—F2D	179.5 (8)
C36B—C31B—C40B—F42B	−163.8 (6)	C6D—C1D—C2D—C3D	1.4 (13)
C36B—C31B—C40B—F43B	75.6 (9)	C6E—C1E—C2E—F2E	177.3 (8)
C40B—C31B—C32B—F33B	74.7 (8)	C6E—C1E—C2E—C3E	−0.1 (13)
C40B—C31B—C32B—F34B	−45.9 (9)	C6F—C1F—C2F—F00S	178.7 (7)
C40B—C31B—C32B—F35B	−165.3 (7)	C6F—C1F—C2F—C3F	1.9 (11)
C40B—C31B—C36B—F37B	−44.0 (9)	C6G—C1G—C2G—F2G	177.7 (8)
C40B—C31B—C36B—F38B	73.6 (9)	C6G—C1G—C2G—C3G	2.9 (13)
C40B—C31B—C36B—F39B	−162.6 (7)	C6H—C1H—C2H—F2H	177.6 (8)
C46B—C45B—C50B—F51B	76.9 (10)	C6H—C1H—C2H—C3H	−2.6 (13)
C46B—C45B—C50B—F52B	−43.6 (10)	C7B—C9—C12—F3B	−3.8 (12)
C46B—C45B—C50B—F53B	−162.6 (8)	C7B—C9—C12—C2B	178.3 (8)
C46B—C45B—C54B—F55B	−161.6 (7)	C7C—C4C—C5C—F5C	−1.5 (11)
C46B—C45B—C54B—F56B	80.1 (10)	C7C—C4C—C5C—C6C	179.1 (8)
C46B—C45B—C54B—F57B	−44.5 (12)	C7D—C4D—C5D—F5D	1.9 (13)
C50B—C45B—C46B—F47B	−44.5 (9)	C7D—C4D—C5D—C6D	−179.2 (8)
C50B—C45B—C46B—F48B	76.4 (9)	C7E—C4E—C5E—F5E	0.2 (12)
C50B—C45B—C46B—F49B	−164.0 (7)	C7E—C4E—C5E—C6E	−179.1 (8)
C50B—C45B—C54B—F55B	−43.9 (10)	C7G—C4G—C5G—F5G	3.7 (11)
C50B—C45B—C54B—F56B	−162.2 (9)	C7G—C4G—C5G—C6G	−175.5 (8)
C50B—C45B—C54B—F57B	73.2 (13)	C7H—C4H—C5H—F5H	−4.0 (11)
C54B—C45B—C46B—F47B	75.6 (9)	C7H—C4H—C5H—C6H	176.0 (8)
C54B—C45B—C46B—F48B	−163.6 (8)	C8—C2—C3—F2	179.6 (7)
C54B—C45B—C46B—F49B	−43.9 (9)	C8—C2—C3—C1	1.1 (12)
C54B—C45B—C50B—F51B	−41.1 (12)	C8A—C3A—C4A—F5A	−166.5 (7)
C54B—C45B—C50B—F52B	−161.6 (8)	C8A—C3A—C4A—F6A	71.6 (9)
C54B—C45B—C50B—F53B	79.4 (10)	C8A—C3A—C4A—F7A	−48.2 (9)
F00S—C2F—C3F—F3F	1.1 (11)	C8A—C3A—C12A—F13A	74.3 (8)
F00S—C2F—C3F—C4F	180.0 (7)	C8A—C3A—C12A—F14A	−41.6 (10)
F1—C2—C3—F2	−0.8 (12)	C8A—C3A—C12A—F15A	−163.5 (7)
F1—C2—C3—C1	−179.3 (7)	C9—C5—C6—F20	−178.5 (8)
F1—C2—C8—C7	178.2 (7)	C9—C5—C6—C4	2.0 (14)
F1—C2—C8—C7A	−3.1 (12)	C12—C2B—C4—F1B	−179.2 (7)
F1A—C1—C2A—F2A	−2.4 (12)	C12—C2B—C4—C6	0.9 (12)
F1A—C1—C2A—C7	179.5 (7)	C12A—C3A—C4A—F5A	74.9 (8)
F1A—C1—C3—F2	2.4 (12)	C12A—C3A—C4A—F6A	−47.1 (9)
F1A—C1—C3—C2	−179.1 (7)	C12A—C3A—C4A—F7A	−166.8 (7)
F1B—C4—C6—F20	−1.1 (12)	C12A—C3A—C8A—F9A	−164.0 (7)
F1B—C4—C6—C5	178.5 (8)	C12A—C3A—C8A—F10A	−46.7 (10)
F1C—C1C—C2C—F2C	−1.4 (12)	C12A—C3A—C8A—F11A	75.4 (10)
F1C—C1C—C2C—C3C	−178.9 (7)	C18A—C17A—C22A—F23A	−45.5 (10)
F1C—C1C—C6C—F6C	−3.4 (13)	C18A—C17A—C22A—F24A	74.4 (13)
F1C—C1C—C6C—C5C	179.6 (8)	C18A—C17A—C22A—F25A	−162.3 (9)
F1D—C1D—C2D—F2D	−0.3 (13)	C18A—C17A—C26A—F27A	77.4 (9)
F1D—C1D—C2D—C3D	−178.4 (8)	C18A—C17A—C26A—F28A	−160.2 (8)
F1D—C1D—C6D—F6D	1.3 (13)	C18A—C17A—C26A—F29A	−40.9 (11)
F1D—C1D—C6D—C5D	178.4 (8)	C22A—C17A—C18A—F19A	−162.5 (8)
F1E—C1E—C2E—F2E	−2.8 (12)	C22A—C17A—C18A—F20A	−38.7 (11)
F1E—C1E—C2E—C3E	179.8 (8)	C22A—C17A—C18A—F21A	80.1 (10)
F1E—C1E—C6E—F6E	−4.0 (13)	C22A—C17A—C26A—F27A	−162.8 (8)
F1E—C1E—C6E—C5E	−179.9 (8)	C22A—C17A—C26A—F28A	−40.4 (10)
F1F—C1F—C2F—F00S	−3.3 (11)	C22A—C17A—C26A—F29A	78.9 (10)
F1F—C1F—C2F—C3F	180.0 (7)	C26A—C17A—C18A—F19A	−43.5 (10)
F1F—C1F—C6F—F6F	2.2 (11)	C26A—C17A—C18A—F20A	80.4 (10)
F1F—C1F—C6F—C5F	−178.8 (7)	C26A—C17A—C18A—F21A	−160.9 (8)
F1G—C1G—C2G—F2G	−3.2 (12)	C26A—C17A—C22A—F23A	−163.9 (8)
F1G—C1G—C2G—C3G	−178.0 (8)	C26A—C17A—C22A—F24A	−44.0 (14)
F1G—C1G—C6G—F6G	−0.9 (13)	C26A—C17A—C22A—F25A	79.3 (11)
F1G—C1G—C6G—C5G	−179.2 (8)	C32A—C31A—C36A—F37A	−38.6 (8)
F1H—C1H—C2H—F2H	−2.2 (12)	C32A—C31A—C36A—F38A	81.3 (8)
F1H—C1H—C2H—C3H	177.6 (8)	C32A—C31A—C36A—F39A	−158.7 (6)
F1H—C1H—C6H—F6H	0.9 (12)	C32A—C31A—C40A—F41A	80.0 (8)
F1H—C1H—C6H—C5H	−179.9 (7)	C32A—C31A—C40A—F42A	−164.0 (6)
F2A—C2A—C7—F3A	3.5 (12)	C32A—C31A—C40A—F43A	−43.2 (10)
F2A—C2A—C7—C8	180.0 (7)	C36A—C31A—C32A—F33A	75.0 (8)
F2B—C2B—C4—F1B	0.7 (11)	C36A—C31A—C32A—F34A	−167.6 (6)
F2B—C2B—C4—C6	−179.2 (7)	C36A—C31A—C32A—F35A	−48.7 (9)
F2B—C2B—C12—F3B	1.7 (12)	C36A—C31A—C40A—F41A	−161.9 (7)
F2B—C2B—C12—C9	179.5 (7)	C36A—C31A—C40A—F42A	−45.9 (8)
F2C—C2C—C3C—F3C	2.2 (12)	C36A—C31A—C40A—F43A	74.9 (9)
F2C—C2C—C3C—C4C	−178.4 (7)	C40A—C31A—C32A—F33A	−165.7 (7)
F2D—C2D—C3D—F3D	−0.7 (13)	C40A—C31A—C32A—F34A	−48.3 (8)
F2D—C2D—C3D—C4D	180.0 (8)	C40A—C31A—C32A—F35A	70.6 (9)
F2E—C2E—C3E—F3E	3.7 (12)	C40A—C31A—C36A—F37A	−157.7 (6)
F2E—C2E—C3E—C4E	−176.1 (7)	C40A—C31A—C36A—F38A	−37.8 (8)
F2G—C2G—C3G—F3G	4.8 (12)	C40A—C31A—C36A—F39A	82.2 (8)
F2G—C2G—C3G—C4G	−179.1 (7)	C46A—C45A—C50A—F51A	−45.8 (11)
F2H—C2H—C3H—F3H	−0.5 (13)	C46A—C45A—C50A—F52A	67.9 (11)
F2H—C2H—C3H—C4H	−176.9 (8)	C46A—C45A—C50A—F53A	−172.0 (8)
F3A—C7—C8—C2	179.2 (7)	C46A—C45A—C54A—F55A	−169.2 (7)
F3A—C7—C8—C7A	0.5 (11)	C46A—C45A—C54A—F56A	−49.3 (10)
F3C—C3C—C4C—C5C	−179.4 (7)	C46A—C45A—C54A—F57A	78.7 (10)
F3C—C3C—C4C—C7C	1.0 (13)	C50A—C45A—C46A—F47A	−47.8 (12)
F3D—C3D—C4D—C5D	−177.0 (8)	C50A—C45A—C46A—F48A	−164.8 (8)
F3D—C3D—C4D—C7D	−0.1 (13)	C50A—C45A—C46A—F49A	75.8 (10)
F3E—C3E—C4E—C5E	177.7 (7)	C50A—C45A—C54A—F55A	−48.9 (10)
F3E—C3E—C4E—C7E	−0.6 (12)	C50A—C45A—C54A—F56A	71.0 (10)
F3F—C3F—C4F—C5F	−177.6 (7)	C50A—C45A—C54A—F57A	−160.9 (8)
F3F—C3F—C4F—C05L	−3.4 (11)	C54A—C45A—C46A—F47A	71.2 (11)
F3G—C3G—C4G—C5G	179.1 (7)	C54A—C45A—C46A—F48A	−45.8 (10)
F3G—C3G—C4G—C7G	−5.6 (11)	C54A—C45A—C46A—F49A	−165.2 (9)
F3H—C3H—C4H—C5H	−178.1 (7)	C54A—C45A—C50A—F51A	−164.0 (8)
F3H—C3H—C4H—C7H	5.2 (12)	C54A—C45A—C50A—F52A	−50.3 (11)
F5C—C5C—C6C—F6C	3.2 (12)	C54A—C45A—C50A—F53A	69.8 (10)
F5C—C5C—C6C—C1C	−179.8 (7)	C26C—C17C—C18C—F21C	−81 (3)
F5D—C5D—C6D—F6D	−2.2 (14)	C26C—C17C—C18C—F20C	37 (3)
F5D—C5D—C6D—C1D	−179.2 (8)	C26C—C17C—C18C—F19C	157 (3)
F5E—C5E—C6E—F6E	3.6 (13)	C26C—C17C—C22C—F23C	−76 (3)
F5E—C5E—C6E—C1E	179.4 (8)	C26C—C17C—C22C—F25C	41 (3)
F5F—C5F—C6F—F6F	−2.3 (12)	C26C—C17C—C22C—F24C	167 (2)
F5F—C5F—C6F—C1F	178.7 (7)	C18C—C17C—C26C—F29C	42 (3)
F5G—C5G—C6G—F6G	1.3 (12)	C18C—C17C—C26C—F27C	162 (2)
F5G—C5G—C6G—C1G	179.5 (7)	C18C—C17C—C26C—F28C	−78 (3)
F5H—C5H—C6H—F6H	0.5 (13)	C18C—C17C—C22C—F23C	163 (3)
F5H—C5H—C6H—C1H	−178.7 (7)	C18C—C17C—C22C—F25C	−80 (3)
F18—C5—C6—F20	2.0 (13)	C18C—C17C—C22C—F24C	46 (3)
F18—C5—C6—C4	−177.5 (8)	C22C—C17C—C26C—F29C	−80 (3)
F18—C5—C9—C7B	0.6 (13)	C22C—C17C—C26C—F27C	41 (3)
F18—C5—C9—C12	177.9 (8)	C22C—C17C—C26C—F28C	161 (3)
O2A—Al1A—O16A—C17A	32.5 (10)	C22C—C17C—C18C—F21C	41 (3)
O2A—Al1A—O30A—C31A	92.4 (9)	C22C—C17C—C18C—F20C	160 (2)
O2A—Al1A—O44A—C45A	162.8 (10)	C22C—C17C—C18C—F19C	−80 (3)
O2A—C3A—C4A—F5A	−43.9 (9)		
