# Supplementary material for: Synthesis and crystal structure of tetra­kis­(perfluoro­benzo­nitrile)­silver(I) tetra­kis­(perfluoro-tert-but­oxy)aluminate(1−): a perfluorinated silver(I) nitrile cation with a perfluorinated weakly coordinating anion
Source: Acta Crystallogr E Crystallogr Commun. 2026 Sep 30;82(Pt 10):1217–20. doi: 10.1107/S205698902600976X (PMC13638011; doi:10.1107/S205698902600976X)

564.6 MHz $^{19}F\{^{1}H\}$ NMR spectrum of $[Ag(NCC_6F_5)_4][Al(OC(CF_3)_3)_4]$ in pentafluorobenzene.

X – peak from pentafluorobenzene solvent;

X – peak from ^{13}C satellites of pentafluorobenzene solvent.

Inset: Expansions of analyte resonances.

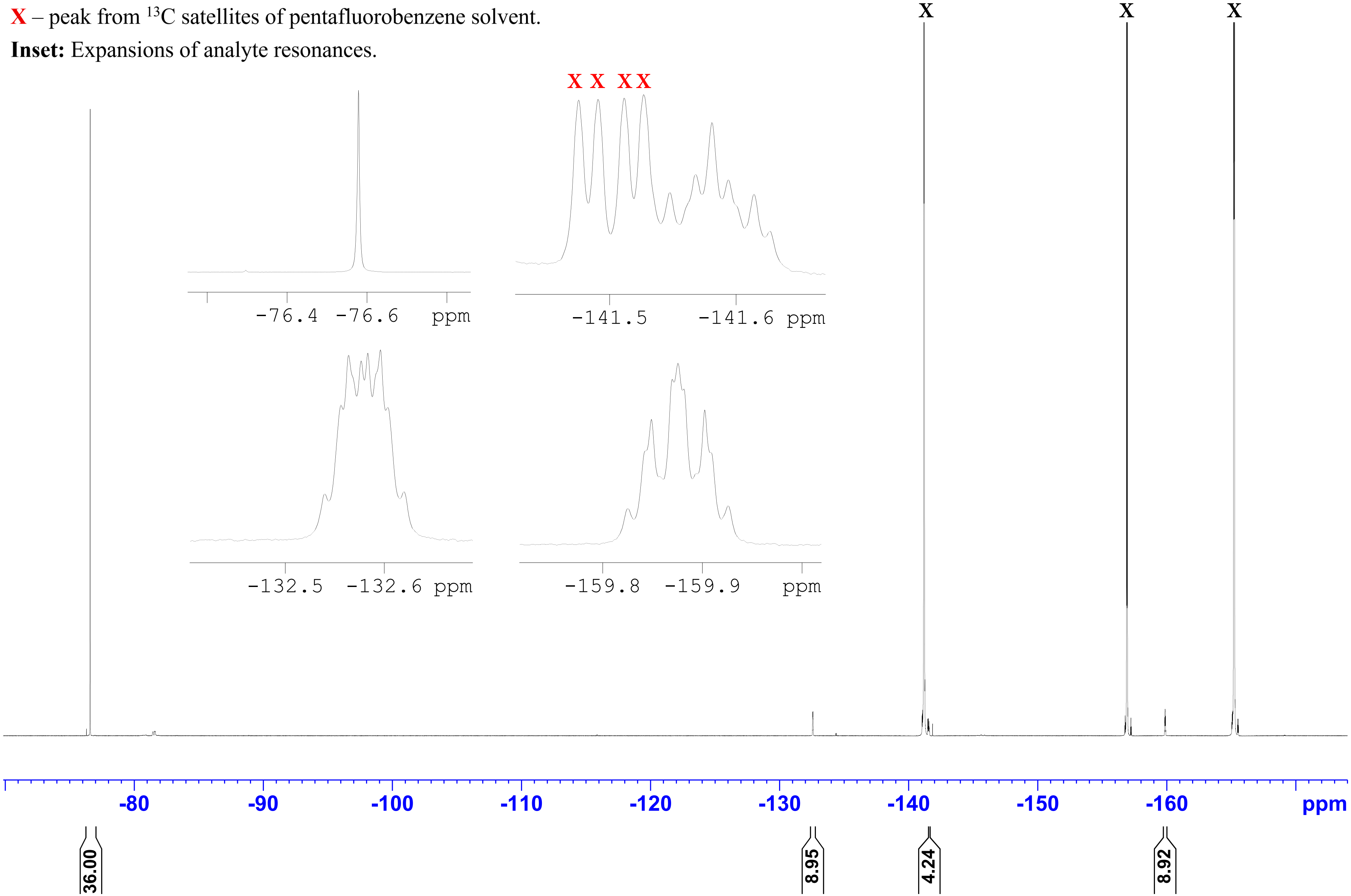

Left: Expansion of peak at -141.6 ppm in $^{19}F\{^{13}C\}$ NMR spectrum of $[Ag(NCC_6F_5)_4][Al(OC(CF_3)_3)_4]$ in pentafluorobenzene.
(^{13}C satellite removed by decoupling)

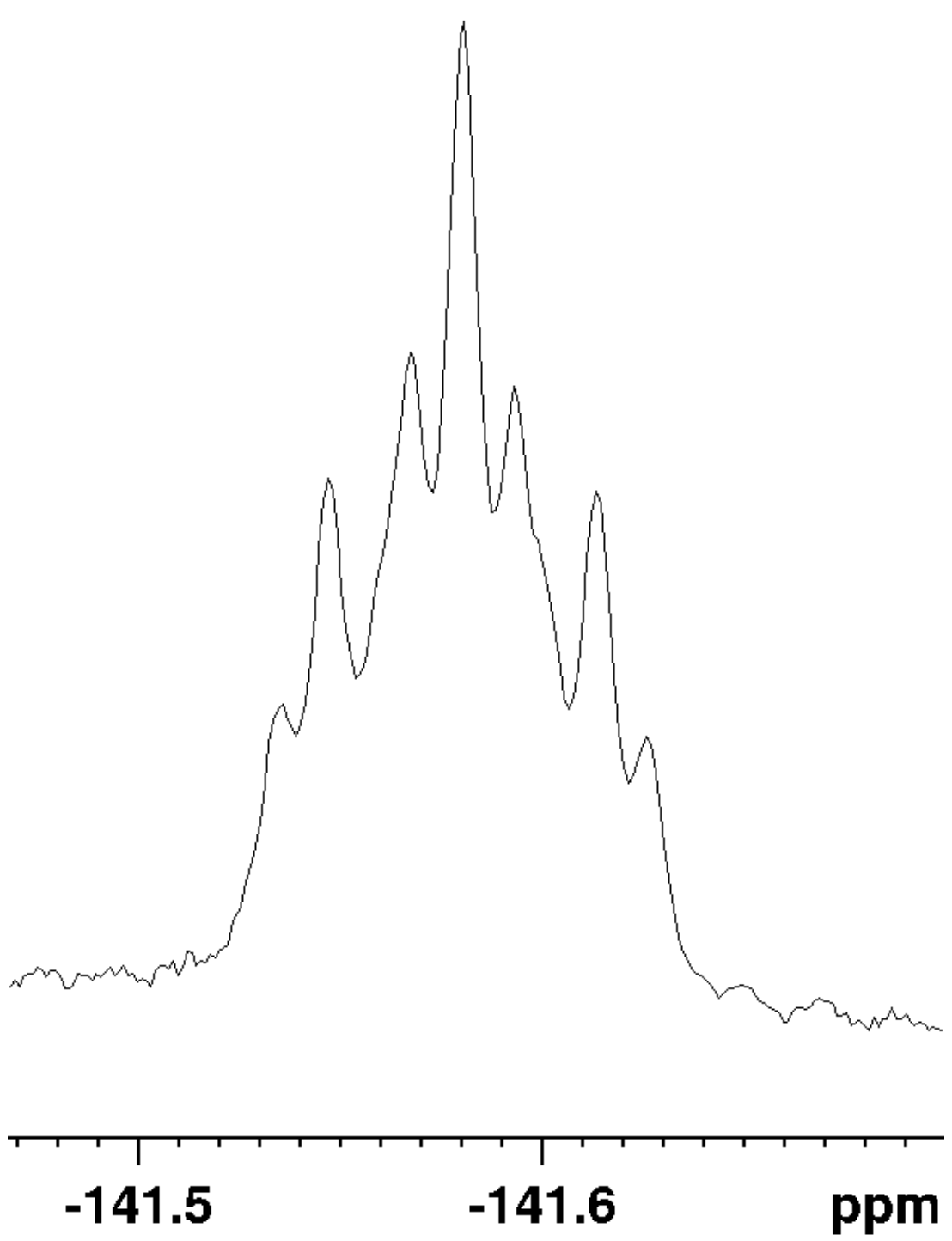

Right: Expansion of 564.6 MHz ^{19}F-19F phase-sensitive COSY NMR spectrum of $[Ag(NCC_6F_5)_4][Al(OC(CF_3)_3)_4]$ in pentafluorobenzene, showing connectivity in the C_6F_5CN unit.

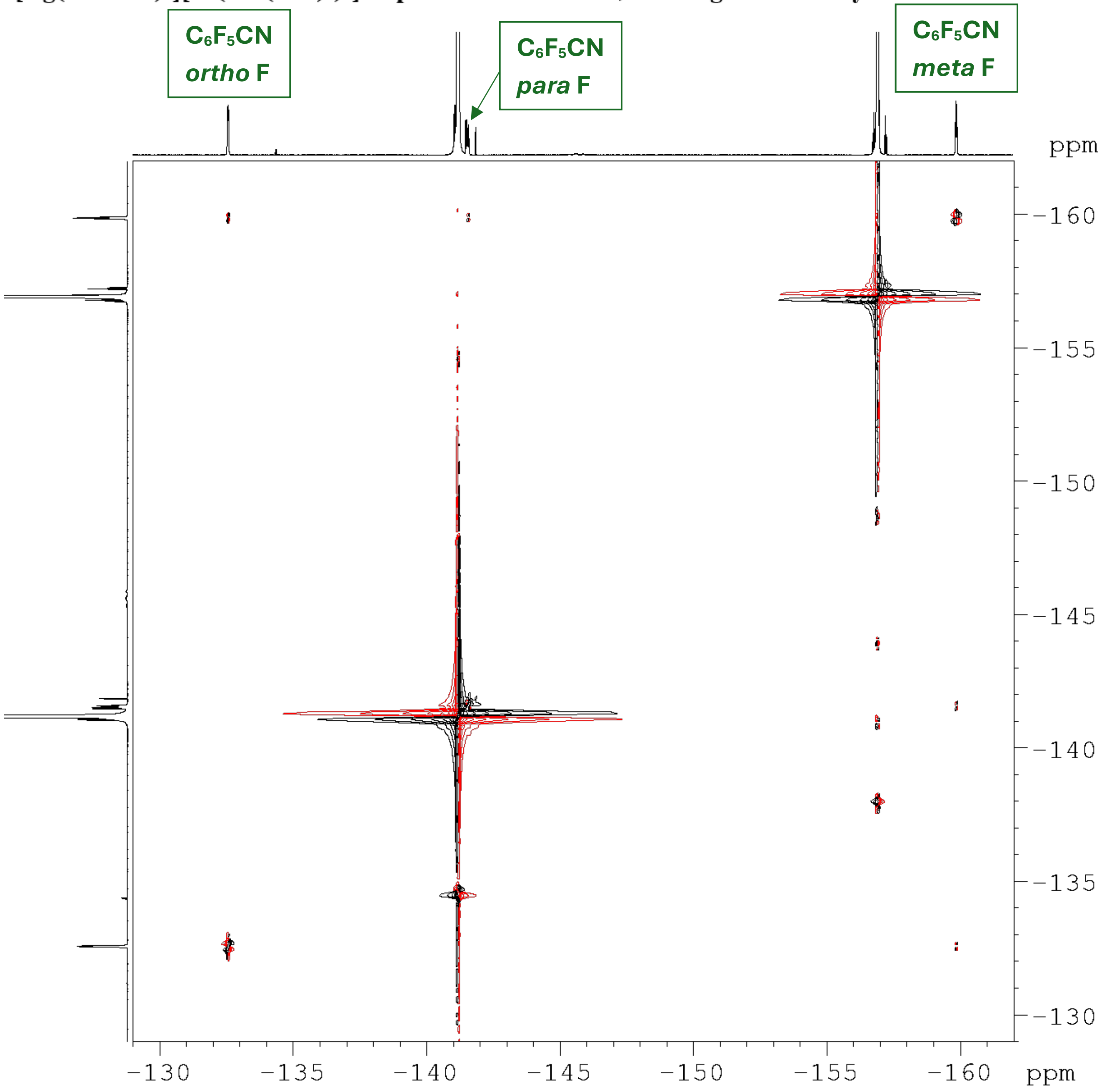

Supplement: Supplementary file 1 [file e-82-01217-sup3.pdf]
